# The origin and impeded dissemination of the DNA phosphorothioation system in prokaryotes

**DOI:** 10.1038/s41467-021-26636-7

**Published:** 2021-11-04

**Authors:** Huahua Jian, Guanpeng Xu, Yi Yi, Yali Hao, Yinzhao Wang, Lei Xiong, Siyuan Wang, Shunzhang Liu, Canxing Meng, Jiahua Wang, Yue Zhang, Chao Chen, Xiaoyuan Feng, Haiwei Luo, Hao Zhang, Xingguo Zhang, Lianrong Wang, Zhijun Wang, Zixin Deng, Xiang Xiao

**Affiliations:** 1 State Key Laboratory of Microbial Metabolism, Joint International Research Laboratory of Metabolic & Development Sciences, School of Life Sciences and Biotechnology, Shanghai Jiao Tong University, Shanghai, China; 2 Southern Marine Science and Engineering Guangdong Laboratory (Zhuhai), Zhuhai, China; 3 Key Laboratory of Combinatorial Biosynthesis and Drug Discovery, Ministry of Education, School of Pharmaceutical Sciences, Wuhan University, Wuhan, China; 4 Simon F. S. Li Marine Science Laboratory, School of Life Sciences, The Chinese University of Hong Kong, Hong Kong, China; 5 Grandomics Biosciences, Wuhan, China

**Keywords:** Microbial ecology, Bacteria, Environmental microbiology

## Abstract

Phosphorothioate (PT) modification by the *dnd* gene cluster is the first identified DNA backbone modification and constitute an epigenetic system with multiple functions, including antioxidant ability, restriction modification, and virus resistance. Despite these advantages for hosting *dnd* systems, they are surprisingly distributed sporadically among contemporary prokaryotic genomes. To address this ecological paradox, we systematically investigate the occurrence and phylogeny of *dnd* systems, and they are suggested to have originated in ancient Cyanobacteria after the Great Oxygenation Event. Interestingly, the occurrence of *dnd* systems and prophages is significantly negatively correlated. Further, we experimentally confirm that PT modification activates the filamentous phage SW1 by altering the binding affinity of repressor and the transcription level of its encoding gene. Competition assays, concurrent epigenomic and transcriptomic sequencing subsequently show that PT modification affects the expression of a variety of metabolic genes, which reduces the competitive fitness of the marine bacterium *Shewanella piezotolerans* WP3. Our findings strongly suggest that a series of negative effects on microorganisms caused by *dnd* systems limit horizontal gene transfer, thus leading to their sporadic distribution. Overall, our study reveals putative evolutionary scenario of the *dnd* system and provides novel insights into the physiological and ecological influences of PT modification.

## Abstract (web summary)

Phosphorothioate (PT) modification by the dnd gene cluster is the first identified DNA backbone modification and has been shown to constitute a multifunctional epigenetic system. Despite a variety of advantages for hosting dnd systems, these systems are surprisingly distributed sporadically among contemporary microbial genomes. To address this ecological paradox, Jian et al. systematically investigated the occurrence and phylogeny of dnd systems in prokaryotes, and provided evidence to suggest that dnd systems have originated in ancient Cyanobacteria (probably Nostocales) after the Great Oxygenation Event.

## Introduction

DNA phosphorothioate (PT) modification is a unique modification of the DNA backbone, in which a non-bridging oxygen atom is swapped with a sulfur atom^1,2^. PT modification is a sequence-selective, stereospecific, post-replicative modification governed by a family of proteins encoded by five genes, termed *dnd* genes (corresponding to the often-observed DNA degradation phenotype during electrophoresis)^3,4^. In *dnd* gene clusters, the *dndA, dndC*, *dndD*, *dndE* genes are essential for PT modification^5^. Among them, DndA acts as a cysteine desulfurase and assembles DndC, which is an iron-sulfur cluster protein that has ATP pyrophosphatase activity and is predicted to have PAPS reductase activity^6,7^. In some cases, DndA can be functionally replaced by the cysteine desulfurase IscS^8^. DndD is believed to provide energy for PT modification^9^, and a DndD homolog known as SpfD in *Pseudomonas fluorescens* Pf0-1 has ATPase activity that is possibly related to DNA structure alterations or nicking during sulfur incorporation^10^. Structural analysis of DndE indicates that this protein is involved in binding nicked dsDNA^11^. Although DndB is not essential for PT modification, its homolog DptB in *Salmonella enterica* serovar Cerro 87 can negatively regulate the transcription of members of the *dptBCDE* gene cluster^12^. Revealed by a co-purification experiment, IscS, DndC, DndD and DndE form a protein complex with the same stoichiometry, and the four proteins assemble into a pipeline according to their gene organization^13^.

Generally, three major physiological functions of PT modifications have been identified. First, PT modification can function as an antioxidant^14–16^. Phosphorothioated DNA affords DNA with reducing ability, reacting with H_2_O_2_, peroxides and hydroxyl radicals in vivo, thus protecting genomic DNA as well as sensitive enzymes from intracellular oxidative damage^14,15^. Correspondingly, the growth range of both the mesophile *Escherichia coli* and the extremophile *Shewanella piezotolerans* WP3 (hereafter referred to as WP3) under multiple stresses increased in response to the antioxidant function of PT modification^16^. Second, PT modification systems can be coupled with PT restriction components (DndFGH), which recognize DNA lacking PT modification in consensus motifs and cleave unmodified DNA, thus forming PT-based restriction-modification (R-M) systems^17,18^. These novel PT R-M systems have been identified in 734 strains and contribute to the defense of microbes against foreign DNA^19^. Finally, DNA PT-based antiviral activities have been recently revealed in some archaea and bacteria, thus expanding the known arsenal of virus resistance systems^20,21^. Moreover, epigenetic regulation of PT has been suggested to occur in *P. fluorescens* Pf0-1, in which the transcriptional efficiency of 4 genes was altered in the presence of PT modifications, according to an in vitro transcriptional assay. This assertion was further supported by characterization of the relationships between two PT-modified sites and the gene expression of *dndB* in *Streptomyces lividans*^22^. Nevertheless, additional convincing evidence, especially the influence of PT modification on the transcription of those non-*dnd* genes, is needed to confirm the epigenetic regulatory function of PT modification.

Since PT modification was first discovered in soil-inhabiting, antibiotic-producing *Streptomyces* species^23^, *dnd* genes have been identified in several prokaryotic genomes and environmental samples^19,24–26^. However, the distribution of these genes is rather sporadic^19,20,26^. In a previous sequence search for the *dnd* system in NCBI nucleotide databases, only 1,349 positive hits were obtained^19^. Moreover, the detection of *dnd* genes in oceanic metagenomes showed that the number of *dnd* genes is often <1% of that of housekeeping genes^26^. In contrast, a survey of 230 diverse bacterial and archaeal genomes revealed DNA methylation in 93% of the genomes and identified 1,459 candidate MTase genes, indicating widespread distribution of DNA methylation in prokaryotes^27^. These results revealed an ecological paradox involving this novel DNA modification system: although PT modifications confer benefits to microbes, why are *dnd* systems only sporadically distributed in a limited number of microorganisms? In actuality, this phenomenon was noticed early, and possible explanations have been proposed in that, despite extensive horizontal gene transfer (HGT), the lability of PT-modified DNA under oxidative stress and its susceptibility to PT-dependent endonucleases has led to widespread but sporadic distribution of PT modifications in bacteria^9^. However, this speculation has not yet been tested. Additionally, a comprehensive evaluation of the occurrence, origin, and evolution of *dnd* systems is lacking, thus severely limiting the ability to resolve this ecological paradox.

Here, to address this paradox, we hypothesized that the laterally transferred *dnd* system would generate a significantly negative effect on the physiology of the recipient microbes, thus limiting the spread of the PT modification system among prokaryotes. In this study, we explore the distribution pattern and ancient ancestor of the *dnd* system in bacterial and archaeal genomes. A variety of experiments are conducted to demonstrate the effect of PT modification on recipient microorganisms after HGT by using the marine bacterium *S. piezotolerans* WP3 and its endogenous bacteriophage SW1 together as a model system. Taken together, we revealthe most likely evolutionary scenario of the *dnd* system and propose that its sporadic distribution is result from the restricted HGT due to transcriptional interference.

## Results

### Distribution of the *dnd* system among prokaryotes

**Fig. 1:**
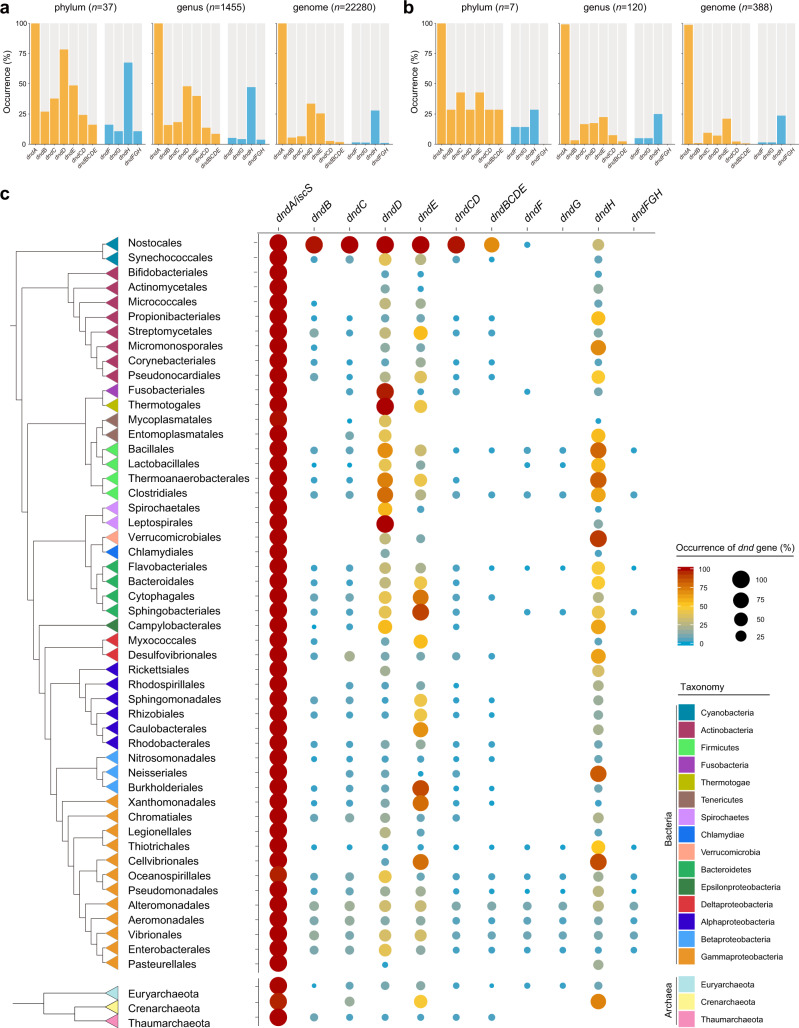
Occurrence and distribution of *dnd* genes and gene clusters among prokaryotes. **a**–**b** Occurrence of *dnd* genes and gene clusters in bacteria and archaea, respectively. The orange and blue bars represent the modification and restriction modules of the *dnd* system, respectively. The total numbers of the analyzed phyla, genera and genomes are shown above each chart. **c** Phylogenetic distribution of *dnd* genes and gene clusters in bacteria (orders) and archaea (phyla). For clarity, only the bacterial orders and archaeal phyla with ≥30 high-quality genomes are shown. The reference phylogeny was reconstructed from the concatenate alignment of 120 and 122 concentrated conserved proteins for bacteria and archaea, respectively. Sericytochromatia (not shown in the diagram) was set as the root lineage for the bacterial phylogenetic tree. The colors of the triangles in the tree show the taxa at the phylum and class (for Proteobacteria) levels. The occurrence of *dnd* genes and gene clusters are quantitatively presented by the colors and size of circles. Source data are provided as a Source Data file.

To comprehensively reveal the distribution of the *dnd* system in prokaryotes, we searched the NCBI Reference Sequence (RefSeq) genome database, which contains the sequences of 22,280 bacterial and 388 archaeal genomes, for different *dnd* genes and gene clusters (Supplementary Data 1). Notably, except for the *dndA*/*isc*S gene, the occurrence of *dnd* genes was substantially limited (Fig. 1). Furthermore, *dndBCDE* gene clusters were present in only 1.82% and 0.77% of the bacterial and archaeal genomes, respectively, indicating the makedly limited distribution of the *dnd* system. Accordingly, the occurrence of PT R components (*dndFGH*) was also limited to 1.09% of the bacterial genomes, and this gene cluster was completely absent in the archaeal genomes (Fig. 1b).

The distribution pattern of the *dnd* system was further demonstrated by coupling the data with those of phylogenetic trees that collapsed at the order and phylum levels for bacteria and archaea, respectively (Fig. 1c and Supplementary Figs. S1 and S2). In addition to that of *dndA*/*isc*S, enrichment of *dndD*, *dndE* and *dndH* genes was noted in several bacterial orders, including Nostocales, Themoanaerobacterales and Sphingobacteriales. However, the presence of *dnd* gene clusters (*dndCD*, *dndBCDE* and *dndFGH*) was substantially limited. Consistent with the findings of a previous report^19^, the occurrence of the PT R component was much lower than that of the M component, indicating the presence of solitary *dndBCDE* gene clusters, especially in Actinobacteria, Alphaproteobacteria, Betaproteobacteria and Gammaproteobacteria (Fig. 1c). In contrast, no enrichment of the *dnd* system was observed in Archaea. Notably, *dnd* genes and gene clusters were significantly enriched in Nostocales in the phylum Cyanobacteria, while the *dndFGH* gene cluster was absent (Fig. 1c). In addition, *dnd* systems that were identified in cyanobacterial genomes had various configurations with different components (Supplementary Fig. S3). The incomplete *dnd* gene cluster and the disorder of the operon structure imply a primitive form of *dnd* systems or that they have undergone vertical evolution for an extremely long time and therefore diversified within the cyanobacterial lineage. Combining this evidence, we supposed that the PT M system initially originated in ancient Cyanobacteria and that cognate PT R components were later developed within the Gammaproteobacteria.

### The *dnd* system probably originated in ancient Cyanobacteria

**Fig. 2:**
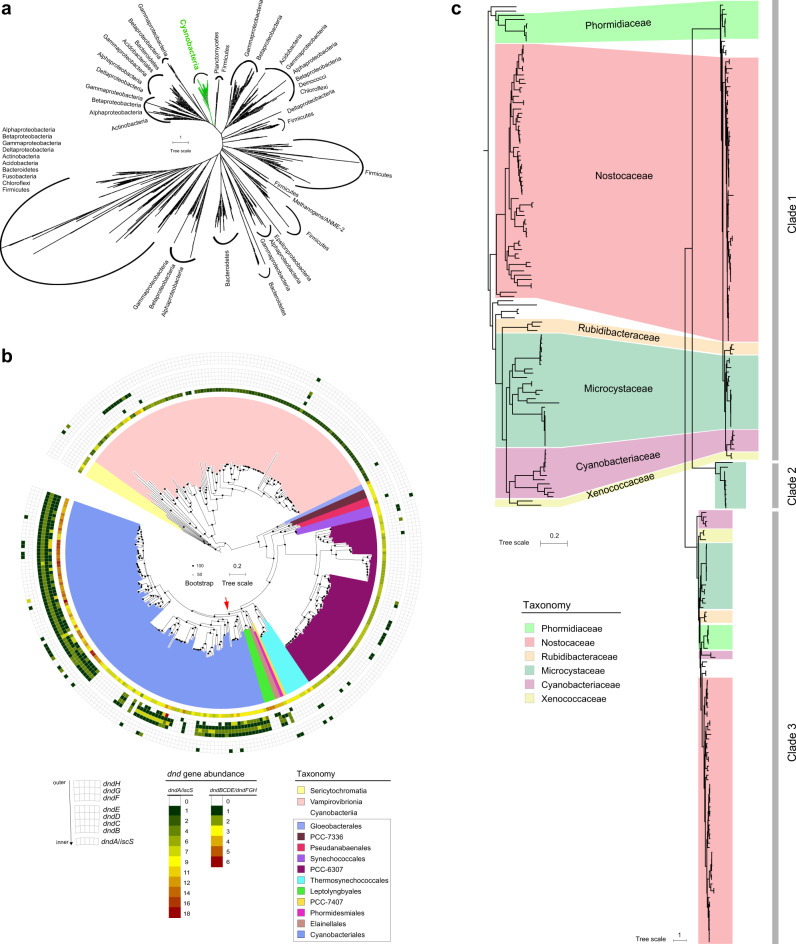
Putative origin of the *dnd* system in Cyanobacteria. **a** Phylogenetic analysis of DndD proteins. The phylogenetic tree is based on the alignments of DndD using MAFFT, filtered with trimAl and constructed by the IQ-Tree method, with the LG + C60 + F + G model and 1,000 bootstrap replicates. The tree branches were classified into different phyla or classes (for Proteobacteria). The green branch belongs to Cyanobacteria, indicating an independent evolutionary path. **b** Phylogenetic distribution of *dnd* genes in Cyanobacteria. The phylogenetic tree was constructed by GTDB-tk, and the taxa are shown in different colors according to GTDB. Sericytochromatia was set as the root lineage. The circular heatmap shows the abundance of *dnd* genes in the corresponding Cyanobacteria genomes. **c** Consistency of the phylogenetic tree of Cyanobacteriales and DndD proteins. The colored linkages refer to the similar phylogenetic positions of the DndD proteins and genomes of those Cyanobacteria. The 3 phylogenetic clades of DndD proteins are indicated by gray bars on the right. Source data are provided as a Source Data file.

The evolutionary history of the *dnd* system was further explored. By using stringent selection criteria for BLASTP (e-value cut off of 10^−20^ and query coverage of 75%), we collected highly reliable sequences of DndD proteins, which are essential components of the *dnd* system and can be used as marker proteins for this system^9^. Phylogenetic analysis indicated that the DndD distribution generally did not match the corresponding species phylogeny (Fig. 2a), suggesting that HGT processes strongly influenced the dissemination of *dnd* genes. However, the DndD from the Cyanobacteria phylum formed a monophyletic group, and these cyanobacterial DndD sequences were not found in the remaining phylogenetic branches (Fig. 2a). Intriguingly, phylogenetic analysis of the DndC protein revealed a similar pattern (Supplementary Fig. S4). Therefore, these results indicated the presence of an independent and conserved evolutionary path in Cyanobacteria.

Next, a Cyanobacteria phylogenomic tree was constructed to further explore the relationship between the evolution of *dnd* genes and Cyanobacteria (Fig. 2b and Supplementary Fig. S5). The results showed that *dnd* systems were absent in Sericytochromatia and Vampirovibrionia, which are sister classes of Cyanobacteriia (GTDB). In contrast, *dnd* gene clusters existed exclusively in Cyanobacteriales but not in other orders (Fig. 2b). The DndD proteins in Cyanobacteriales formed two major and one minor phylogenetic clade (Fig. 2c and Supplementary Fig. S6). Among them, the phylogenetic positions of one of the major clades (clade 1) in the well-supported tree were highly consistent with the positions in the enlarged Cyanobacteriales phylogenomic tree (Fig. 2c), suggesting that the *dnd* systems were vertically inherited in the Cyanobacteriales order. Based on this evidence, we propose that the ancestor of Cyanobacteriales strains could be considered a candidate host in which the *dnd* system originated.

### The occurrence of *dnd* systems is negatively correlated with prophages

**Fig. 3:**
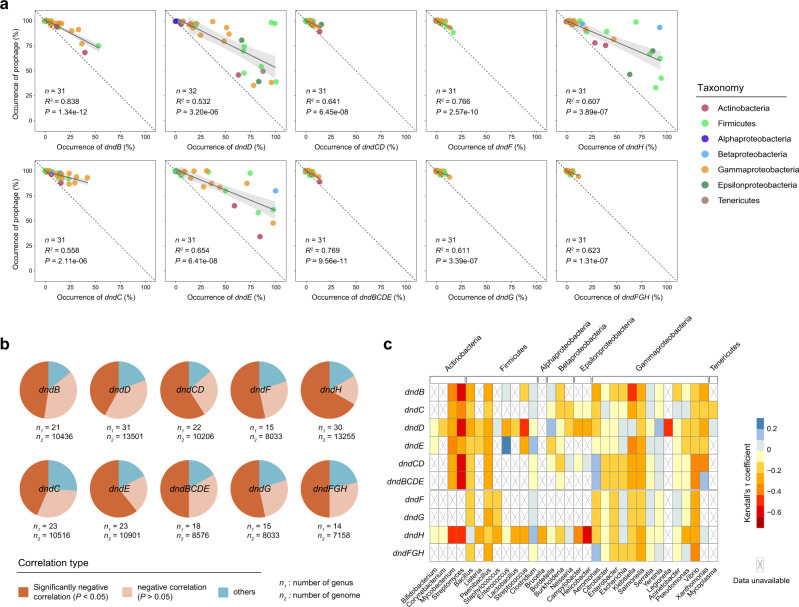
Negative correlations between the occurrence of *dnd* genes/gene clusters and prophages are predominant in bacteria. **a** Correlation analysis between the occurrence of *dnd* genes/gene clusters and prophages among different genera. The black solid line in each sub-plot refers to the best fitting, and the gray shadow displays the 95% confidence interval from linear regressions. The dashed lines depict the 1:1 linear relationship. The number of analyzed genera (*n*), *R*^2^ values and *P* values of linear regressions are shown in each sub-plot. Each circle represents a single genus, and is depicted in the color representing the taxonomy at the phylum and class levels (for Proteobacteria). **b**, **c** Correlation analysis between the occurrence of *dnd* genes/gene clusters and prophages in thoroughly sequenced bacterial genera (with ≥100 genomes). The correlation type is identified based on Kendall’s τ coefficient and significance values. *n*_*1*_ and *n*_*2*_ below each pie chart **b** refer to the number of genera and included genomes, respectively. The heatmap **c** shows Kendall’s τ coefficient, and the crosses indicate statistical unachievability due to a small sample size (number of genomes <30) or the complete absence of *dnd* genes/gene clusters or prophages in the corresponding genus. The taxonomy of the genera and phyla/classes are shown below and above the heatmap, respectively. Source data are provided as a Source Data file.

We subsequently aimed to reveal the causes of the sporadic distribution of the *dnd* system. Interestingly, we occasionally noticed that prophages were not detected in bacterial genomes that harbored *dnd* gene clusters (Supplementary Fig. S7). This phenomenon led us to assess the relationship between the occurrence of *dnd* systems and prophages in a large dataset. The sequences of all publicly available bacterial genomes in the NCBI RefSeq database were downloaded and analyzed. To facilitate statistical analysis, only the 32 genera that contained >100 genomes of high quality were included in the following analysis. We searched for prophages in 13,916 bacterial genomes belonging to these 32 genera, and 24,720 prophages were identified in 7,549 genomes (Supplementary Data 2). Notably, the occurrence of all *dnd* genes and gene clusters was significantly negatively correlated (linear regression *R*^*2*^ > 0.5, *P* < 0.001; Pearson coefficient < −0.7, *P* < 0.001) with the occurrence of prophages (Fig. 3a and Supplementary Table S1). This conclusion was further supported by a similar analysis among different species (Supplementary Fig. S8 and Table S1). Generally, individual *dnd* genes generally have a wider occurrence range than gene clusters. Remarkably, in contrast to the high prevalence of prophages in bacteria, the occurrence of *dnd* gene clusters was limited to a low amount (Fig. 3a).

To further quantify the pervasiveness of the negative association between prophages and the *dnd* system, we calculated Kendall’s τ coefficient between the abundance of prophages and *dnd* genes/gene clusters in all genomes for each genus (Supplementary Data 3). The results revealed negative correlations in the majority of these genera (Fig. 3b). Notably, the abundance of prophages and PT M systems were significantly negatively correlated (*P* < 0.05) in 50% of the analyzed genera, which contained 8,578 genomes. Again, a similar correlation was detected between the prophages and PT R system. Specifically, the number of prophages and *dndBCDE* was most strongly negatively correlated in *Streptomyces* (Kendall’s τ coefficient = −0.61, *P* = 3.25E-07) and *Vibrio* (Kendall’s τ coefficient = −0.34, *P* = 3.82E-08), and the number of prophages was also strongly negatively correlated with *dndFGH* in *Vibrio* (Kendall’s τ coefficient = −0.33, *P* = 1.26E-07) (Fig. 3c). Moreover, the association between prophages and the *dnd* system was further measured at the species level, and similar results were obtained (Supplementary Data 3). Overall, these results clearly indicated that the occurrence of *dnd* systems is negatively correlated with prophages in bacterial genomes. We therefore hypothesized that horizontally transferred *dnd* genes and the accompanying PT modifications may activate prophages, and the subsequent cell lysis and decrease in microbial abundance would significantly inhibit *dnd* system dissemination among prokaryotes.

### PT modifications activate prophage by altering the binding affinity of phage repressor and the transcription level of its encoding gene

**Fig. 4:**
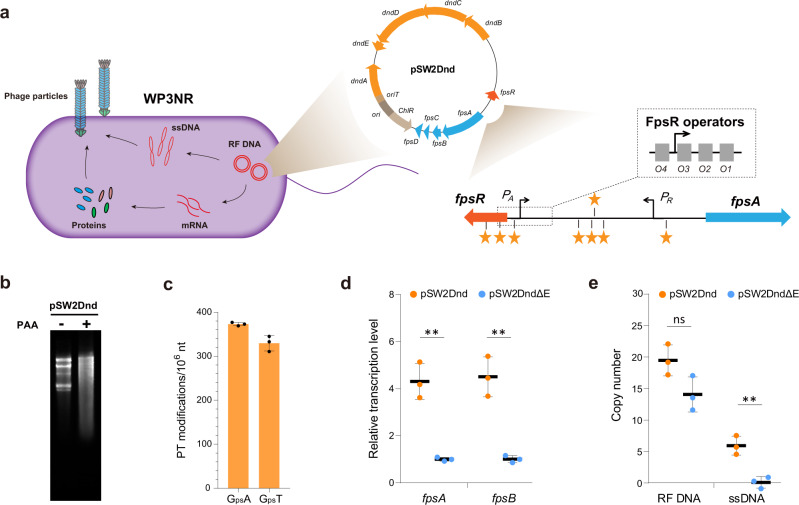
PT modification influences the gene transcription and DNA replication of phage SW1. **a** Schematic representation of the WP3NR- pSW2Dnd system for investigating the effect of PT modification. The double-stranded replicative forms of DNA (RF DNA), single-stranded DNA (ssDNA) and mRNA which were quantified via qPCR, are indicated in red. The promoters of *fpsA* (*P*_*A*_) and *fpsR* (*P*_*R*_) are responsible for the transcription of the SW1 structural genes *fpsA-H* and the regulator gene *fpsR*, respectively. The phage-encoded repressor FpsR binds to four operators located in *P*_*A*_ and the intergenic region between *fpsA* and *fpsR*, thus functioning as the determinant of the genetic switch of SW1. The yellow stars indicate the putative PT modification sites. For clarity, the genes and regulatory elements are not drawn to scale. **b** Cleavage detection of PT modification in pSW2Dnd. Agarose gel showing the effect of treating pSW2Dnd from WP3NR/Dnd with PAA–TAE buffer. “+” and “−” represent treated and untreated plasmids, respectively. **c** Quantification of PT modifications (G_ps_A and G_ps_T) in pSW2Dnd. The data represent the mean ± s.d. and are based on three biologically independent samples. **d** Relative transcription levels (RTLs) of SW1 genes in the WP3NR/Dnd and WP3NR/DndΔE strains. **e** RF DNA and ssDNA copy numbers of pSW2Dnd and pSW2DndΔE. Data are represented as mean ± s.d.. and based on three biologically independent samples. The significances were analyzed by two-sided unpaired Student’s *t* test. Specifically, *P* = 0.0017 (^∗∗^) for *fpsA* and *P* = 0.0022 (^∗∗^) for *fpsB* of pSW2Dnd vs pSW2DndΔE; *P* = 0.0643 (ns, not significant) for RF DNA and *P* = 0.0046 (^∗∗^) for ssDNA of pSW2Dnd vs pSW2DndΔE. Source data are provided as a Source Data file.

To address the aforementioned hypothesis, the influences of PT modification on prophages were assessed in a model system that was derived from the temperate phage SW1 and its bacterial host *S. piezotolerans* WP3 and has previously been utilized to explore the physiological influence of PT modification^16,28^. In particular, the whole *dnd* gene cluster (*dndABCDE*) from *S. enterica* serovar Cerro 87 was cloned into a pSW2 shutter vector to generate pSW2Dnd (Fig. 4a). Correspondingly, eight putative PT modification motifs in the *fpsR-fpsA* intergenic region were identified according to the conserved PT modification sites (5′-G_ps_AAC-3′/5′-G_ps_TTC-3′) of *S. enterica* serovar Cerro 87^29^. Furthermore, two of these motifs were located within FpsR operators, which are responsible for the genetic switch of phage activation^30^. Exposure of pSW2Dnd from WP3NR to peracetic acid (PAA) and Tris-acetate EDTA (TAE) buffer caused extensive DNA cleavage (Fig. 4b), which is the typical in vitro characteristic of PT-modified DNA^3^. DNA PT modifications were then quantitatively analyzed; the frequencies of d(G_ps_A) and d(G_ps_T) modifications were 373 ± 3 and 329 ± 14/10^6^ nt, respectively, which is equivalent to 11 d(G_ps_A) and 9 d(G_ps_T) modifications in every pSW2Dnd plasmid (Fig. 4c). Taken together, the above-described data demonstrated that the transferred *dnd* gene cluster was functional in WP3NR cells.

The relative transcription levels (RTLs) of SW1 genes *fpsA* and *fpsB* in pSW2Dnd were subsequently assessed by comparison with those in pSW2DndΔE (Supplementary Fig. S9), which lacked a *dndE* gene and thus did not show sensitivity to cleavage by PAA-TAE^31^. These derivatives of pSW2 vectors remain stable during cultivation and could therefore be used to accurately evaluate the effects of PT modification on transcription (Supplementary Fig. S10). Interestingly, the RTLs of *fpsA* and *fpsB* were significantly higher in pSW2Dnd than in pSW2DndΔE (Fig. 4d), indicating that PT modification influenced the transcription of SW1. Here, the possibility that this differential expression resulted from the SOS response as previously reported in *S. enterica* was ruled out, as neither cell elongation nor upregulated expression of the key SOS genes *recA* and *lexA* (both of which are typical features of the SOS response^32^) was observed in the PT-modified strain (Supplementary Fig. S11). Additionally, we also excluded the possibility of differences due to PT-specific endonucleases, because neither a homologous gene of *dndFGH* was found in the WP3 genome nor differences in nuclease activity were detected in different WP3NR strains (Supplementary Fig. S12). We therefore were convinced that PT modification directly caused a change in phage SW1 transcription.

To further assess whether PT modification influenced the life cycle of SW1, the copy numbers of double-stranded replicative form (RF) DNA and the single-stranded (ss) DNA of pSW2Dnd and pSW2DndΔE were quantified. The data showed that ssDNA substantially accumulated after PT modification, indicating that the genetic switch of SW1 was activated during this situation (Fig. 4e). To confirm the effect of PT modification on the bacteriophage, we transferred pSW2Dnd and pSW2DndΔE into WP3ΔRE, which is a non-restricting WP3 strain harboring intact phage SW1^33^. As expected, the RTLs of *fpsA* and *fpsB* significantly increased in WP3ΔRE/Dnd compared with WP3ΔRE/DndΔE (Supplementary Fig. S13a). Notably, the ssDNA of SW1 accumulated to high levels in the PT-modified strain WP3ΔRE/Dnd (Supplementary Fig. S13b). Therefore, our results suggested that PT modification functions as an anti-repressor, activating phage gene transcription and DNA replication.

**Fig. 5:**
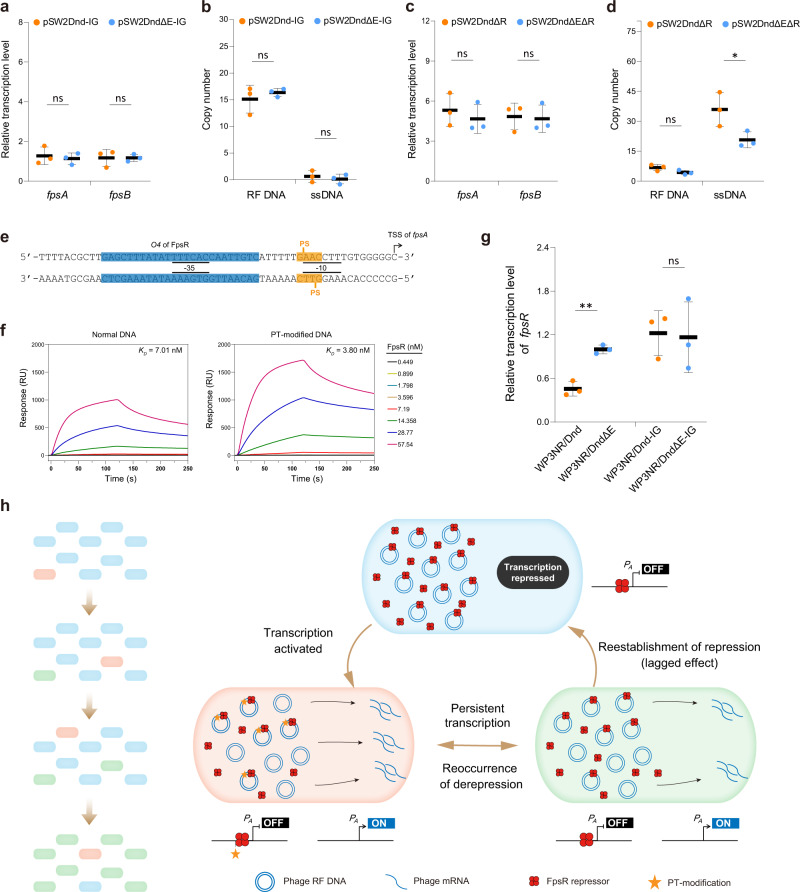
PT modification activates phage SW1 by influencing the repressor FpsR. **a** RTLs of SW1 genes in the WP3NR/Dnd and WP3NR/DndΔE strains with point mutations in PT modification sites. **b** RTLs of SW1 genes in the WP3NR/Dnd and WP3NR/DndΔE strains with *fpsR* gene deletion. **c** RF DNA and ssDNA copy numbers of pSW2Dnd-IG and pSW2DndΔE-IG. **d** RF DNA and ssDNA copy numbers of pSW2DndΔR and pSW2DndΔEΔR. Data are represented as mean ± s.d. and based on three biologically independent samples. The significances were analyzed by two-sided unpaired Student’s *t* test. Specifically, *P* = 0.6671 (ns) for *fpsA* and *P* = 0.9976 (ns) for *fpsB* of pSW2Dnd-IG vs pSW2DndΔE-IG; *P* = 0.5763 (ns) for RF DNA and *P* = 0.4729 (ns) for pSW2Dnd-IG vs pSW2DndΔE-IG; *P* = 0.5352 (ns) for *fpsA* and *P* = 0.8514 (ns) for *fpsB* of pSW2DndΔR vs pSW2DndΔEΔR; *P* = 0.0927 (ns) for RF DNA and *P* = 0.0486 (^∗^) for ssDNA of pSW2DndΔR vs pSW2DndΔEΔR. **e** Chemically synthesized PT-modified DNA probe for SPR assays. The transcription start sites of *fpsA* are marked with angled arrows. The −35/−10 consensus elements of the *fpsA* promoter are underlined with solid lines. The PT modification sites and FpsR operator site (*O4*) are highlighted in yellow and blue, respectively. **f**. SPR sensorgrams of the binding of FpsR to normal and PT-modified DNA. The FpsR protein was injected over the sensor chip at concentrations ranging from 0.449 to 57.54 nM, and the DNA-binding activity is given in response units (RU). The *K*_*D*_ of FpsR binding was subsequently determined. **g** RTLs of the *fpsR* gene in different WP3NR strains. Data are represented as mean ± s.d. and based on three biologically independent samples. The significances were analyzed by two-sided unpaired Student’s *t* test. Specifically, *P* = 0.0013 (^∗∗^) and *P* = 0.8737 (ns) for *fpsR* in WP3NR/Dnd vs WP3NR/DndΔE and WP3NR/Dnd-IG vs WP3NR/DndΔE-IG, respectively. **h** Proposed underlying mechanism responsible for the derepression of phage SW1 gene transcription by PT modification. In the WP3NR/Dnd strain, the significantly reduced amount of FpsR preferentially binds to PT-modified DNA instead of normal DNA due to a higher binding affinity, thereby releasing a proportion of the phage promoter to be derepressed. Moreover, as the PT motifs are dynamically and partially modified, a “lagged effect” due to the different time requirements for the change in PT modification status and the reestablishment of prophage repression, may also contribute to the derepression of phage gene transcription. Source data are provided as a Source Data file.

The binding of the phage-borne repressor FpsR to its cognate operator is the key controlling factor for the SW1 genetic switch^30^. To further explore the underlying mechanism through which PT modification affects phage gene expression, 8 PT modification motifs located within the *fpsR-fpsA* intergenic region were point mutated in pSW2Dnd and pSW2DndΔE to generate two vectors: pSW2Dnd-IG and pSW2DndΔE-IG (Supplementary Fig. S14). As expected, subsequent real-time qPCR (RT-qPCR) showed that there were no significant differences in the RTLs of SW1 genes and in the copy number of phage ssDNA after the PT modification motifs were mutated (Fig. 5a).

We then examined whether the activation of phage gene transcription was due to the effects of PT modification on the activity of RNA polymerase or FpsR. First, the possibility of influence on RNA polymerase was ruled out by an in vitro transcription assay, as no significant difference in RNA production was detected (Supplementary Fig. S15). To test another possibility, we constructed two vectors, pSW2DndΔR and pSW2DndΔEΔR, in which the *fpsR* gene was deleted in-frame from pSW2Dnd and pSW2DndΔE, respectively (Supplementary Fig. S9). The RTLs of *fpsA* and *fpsB* significantly increased in these two vectors, but there was no significant difference in the levels between pSW2DndΔR and pSW2DndΔEΔR (Fig. 5c). Moreover, significant ssDNA production was observed in both vectors because of the derepressing effect of *fpsR* deletion (Fig. 5d). Taken together, these results suggest that PT modification influences the regulatory function of FpsR, thus affecting phage gene transcription.

To further test whether DNA PT modification affected the binding affinity of FpsR, PT-modified DNA and normal DNA probes that covered the *fpsA* promoter were chemically synthesized (Fig. 5e). Notably, one of the FpsR operators (*O4*) and a PT modification site were located within the −35 and −10 regions, respectively, of the *fpsA* promoter. A real-time surface plasmon resonance (SPR) experiment was then performed (Fig. 5f), and the equilibrium dissociation constants (*K*_*D*_) obtained from the curve fit were 7.01 nM and 3.80 nM for FpsR binding with normal and PT-modified DNA, respectively (Fig. 5f and Supplementary Table S2). Besides that, the RTLs of the *fpsR* gene were significantly decreased after PT modification (Fig. 5g), indicating a lower amount of FpsR in the PT-modified WP3 strains. These data thus support a model in which PT modification substantially increased the binding affinity of FpsR to the *fpsA* promoter, and decreased the amount of FpsR protein, thereby relieving the repression of phage gene transcription because of the competitive binding of operators with different statuses of modification involving FpsR (Fig. 5h). Moreover, considering the dynamic nature of PT motifications^9^, we proposed that a “lagged effect” resulting from the different time requirements for the change in PT modification status and the reestablishment of repression probably also contributed to the derepression process.

### Horizontal transfer of the *dnd* system reduces competitive fitness by perturbing gene transcription

**Fig. 6:**
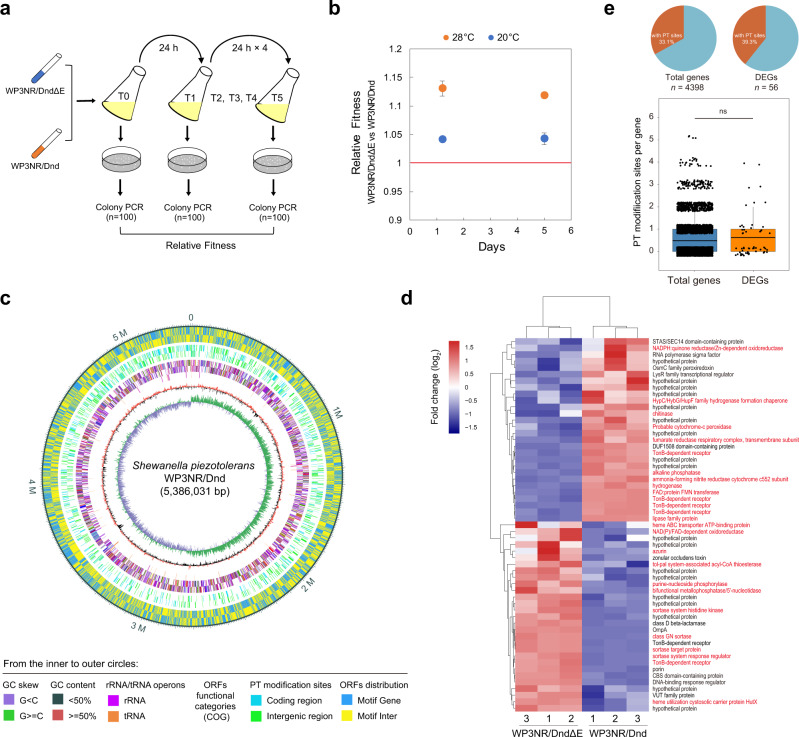
The horizontally transferred *dnd* gene cluster substantially reduces the fitness of the deep-sea bacterium *S. piezotolerans* WP3. **a** Experimental scheme of competition experiments performed. Co-culture competition assays were performed in 2216E media at 28 °C and 20 °C. The details can be found in the Methods section. **b** Fitness measurements of two WP3 strains (WP3NR/DndΔE vs. WP3NR/Dnd) at different temperatures. The red line indicates a relative fitness of 1 (no fitness difference). Data are represented as mean ± s.d. and based on three biologically independent samples. **c** Detection of PT sites by SMRT sequencing across the genome of *S. piezotolerans* WP3NR/Dnd. The annotations from the inner to outer circles are indicated below the diagram. **d** Comparison of gene expression profiles in WP3NR/Dnd relative to WP3NR/DndΔE. The RNA-seq data represent three biologically independent samples for each strain, and the data were hierarchically clustered. Normalized differential expression is shown in the heat map according to the scale bar (log_2_ scale) from most upregulated (red) to most downregulated (blue). The DEGs involved in metabolic function are indicated in red in the right panel. **e** Distribution of PT sites within the DEGs. The genomic PT profile and transcriptome data were combined to reveal the relationship between them. Boxplot components: center line, median; box limits, upper and lower quartiles; whiskers, 1.5× interquartile range. The significance was analyzed by two-sided unpaired Student’s *t* test (ns, not significant, *P* = 0.3292). Source data are provided as a Source Data file.

To further elucidate the reason for the sporadic distribution of the *dnd* system, we questioned whether exogenous introduction of the *dnd* gene cluster confers competitive disadvantages to a non-PT bacterium. To this end, a competition assay was performed via coculture of WP3NR/Dnd and WP3NR/DndΔE at 20 °C (the optimum growth temperature of WP3) (Fig. 6a). In the starting culture (T0), WP3NR/Dnd accounted for 52% of the population. After 1-day- and 5-day-long incubation periods, the percentage of WP3 PT strains in the population decreased to 48 and 31%, respectively (Supplementary Table S3). The relative fitness values of the WP3NR/DndΔE vs WP3NR/Dnd strains from T1 vs. T0, T5 vs. T0 and T5 vs. T1 were 1.038 ± 0.022, 1.041 ± 0.003 and 1.043 ± 0.010, respectively (Fig. 6b), indicating decreased competitive fitness due to the *dnd* system. The competition experiment was also performed at 28 °C (the maximal growth temperature of WP3). Similarly, the coculture was dominated by the non-PT strain (93%) after 5 days of cultivation (Supplementary Table S3), and the calculated relative fitness values from T1 vs. T0, T5 vs. T0 and T5 vs. T1 were 1.180 ± 0.047, 1.131 ± 0.013 and 1.119 ± 0.005, respectively (Fig. 6b), indicating that the PT system conferred a stronger competitive disadvantage to WP3 under high temperature stress.

Next, we concurrently performed genomic PT mapping and global transcriptome analysis to explore why the horizontally transferred *dnd* system reduced the competitive fitness of the marine bacterium *S. piezotolerans* WP3. Single-molecule real-time (SMRT) sequencing was used, which led to the identification of 2,254 PT modification sites within the WP3NR genome (Fig. 6c and Supplementary Data 4)-1,203 5′-G_ps_AAC-3′ sites and 1,329 5′-G_ps_TTC-3′ sites, which accounted for 4.05% and 4.48%, respectively, of the total number of PT motifs in the genome. This coincides with the nature of PT modification, which has been revealed to be partial or incomplete^9,29^. Moreover, the calculated frequencies of d(G_ps_A) (223.36/10^6^ nt) and d(G_ps_T) (246.75/10^6^ nt) modification in the WP3NR/Dnd genome were in accordance with the mass spectrometric evidence (200 ± 7 and 221 ± 24/10^6^ nt for G_ps_A and G_ps_T, respectively) (Supplementary Fig. S16), and both were comparable to the previously reported modification frequencies in *S. enterica* serovar Cerro 87^34^. Additionally, d(G_ps_A) and d(G_ps_T) modifications were identified in the coding regions of 934 and 1,018 genes, respectively, and were evenly distributed throughout the genome (Fig. 6c).

Transcriptomic analysis was performed to investigate the influence of PT modification on the gene transcription profile of WP3NR. The RNA sequencing (RNA-seq) data were validated via RT-qPCR analysis, which showed a strong correlation coefficient (*R*^*2*^ = 0.9653) (Supplementary Fig. S17), indicating that the transcriptomic data were reliable and could be used for follow-up analysis. Again, the possibility that this differential expression resulted from the SOS response was ruled out, as neither cell elongation nor upregulated expression of *recA*/*lexA* was detected in the PT-modified strain (Supplementary Fig. S18 and Table S4). Overall, 57 genes were found to be differentially expressed (FDR < 0.05 and FC > 2) between WP3NR/Dnd and WP3NR/DndΔE (Fig. 6d and Supplementary Table S4). Notably, hierarchical clustering analysis indicated that enriched differentially expressed genes (DEGs) (26/57) were associated mainly with cellular metabolic functions according to classification via the KEGG database (Fig. 6d), probably explaining the decreased fitness of the PT-modified strain. Further coupled analysis revealed that although the proportion of DEGs containing PT sites was slightly higher than that of other genes (39.3% vs. 33.1%), the average number of PT sites they contain did not significantly differ from that of other genes (Fig. 6e), indicating that transcription alteration of and PT modification in a single gene were likely not directly correlated.

Regarding the substantial competitive disadvantage of WP3NR/Dnd at high temperature (28 °C), we speculated that PT modification perturbed the transcription of genes encoding heat-shock proteins (HSPs), which include chaperones and proteases and are essential for overcoming changes that involve protein denaturation during thermal stress^35^. To address this possibility, eight HSPs were chosen as representatives, and the RTLs of their encoding genes at different temperatures were measured in the WP3NR/Dnd and WP3NR/DndΔE strains. The expression of HSP-encoding genes in both strains was significantly upregulated at 28 °C compared with 20 °C. However, the extent of the upregulation of these genes in the PT-modified strain was significantly less than that in the control strains (Supplementary Fig. S19). Specifically, the transcript levels of the *hslU*, *hsp70* and *dnaJ* genes were >10-fold lower in WP3NR/Dnd than in WP3NR/DndΔE. These results strongly suggested that PT modification interferes with the normal induction of the heat-shock response, resulting in a competitive disadvantage for WP3NR at high temperature (Fig. 6b).

## Discussion

**Fig. 7:**
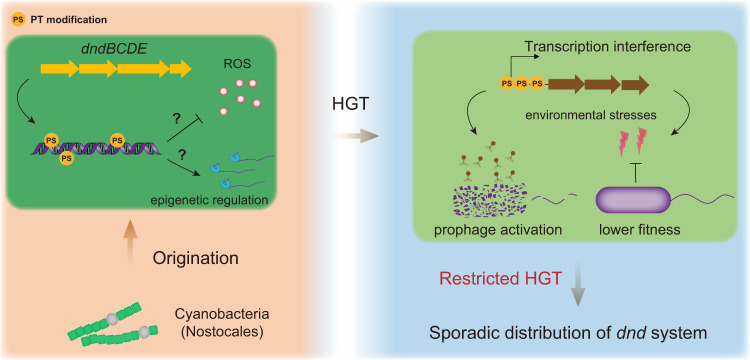
Schematic representation of the hypothetical evolutionary scenario of the *dnd* system. The *dnd* system with a primordial configuration originated in ancient Cyanobacteria (probably Nostocales). The original *dnd* system is thought to have dealt with the emergence of reactive oxygen species (ROS) after the GOE. Alternatively, it may evolve first as an epigenetic system for gene regulation. The decreased fitness (including induction of inherent prophages and a weakened ability to respond to environmental stresses) resulting from transcription interference by PT modification would subsequently lead to a substantial restriction of HGT of the *dnd* system between microorganisms, thereby causing their sporadic distribution in present environments.

As the earliest oxygen-producing life forms, Cyanobacteria are thought to be responsible for the steady increase in oxygen concentration on Earth by oxygenic photosynthesis^36,37^. The origins of Cyanobacteria can be dated back to 2.7 billion years ago^37^, and these organisms have been considered the key players in the Great Oxygenation Event (GOE) on Earth ~2.33 billion years ago^38^. Additionally, sulfur-based metabolism has been suggested to be very ancient, because sulfur-metabolizing cells have been preserved in 3.4-billion-year-old microfossils^39,40^. In this study, evolutionary analysis indicated that the *dnd* system likely originated from ancient Cyanobacteriales, probably the ancestors of Nostocaceae (Nostocales, according to NCBI taxonomy). Nostocales belong to category IV of the subsection of Cyanobacteria, and their most notable feature is the ability to form heterocysts for nitrogen fixation^37,41^. Fossil evidence indicates that their existence and distribution fit well with the timing of the end of the GOE over a long period of geological history (2,100–720 Ma)^42,43^. Consistent with this, *dnd* systems were absent in Sericytochromatia and Vampirovibrionia (Fig. 2b), which are sister groups of Cyanobacteria, and their differentiation from Cyanobacteria occurred before the GOE^44^. This evidence thus supports the inference that the *dnd* system originated after the GOE. As the PT R systems are absent in Cyanobacteria (Figs. 1c and 2b), it is highly probable that the *dnd* gene clusters were originally used as antioxidant systems, while their coupling with the PT M component occurred later. In accordance with this speculation, the distribution of antioxidant-related genes in Cyanobacteria was found to be consistent with that of the *dnd* system (Supplementary Fig. S20). Taken together, these results suggest that the *dnd* system formed after the GOE and that this system may have been initially used by Cyanobacteria to combat oxidative stress caused by rising oxygen concentrations on Earth. Meanwhile, considering the analogy between the orphan PT and methylation-based modification systems, the possibility that the *dnd* system evolved first for epigenetic regulation cannot currently be excluded (Fig. 7).

Intriguingly, *dnd* gene clusters were frequently identified within mobile genetic elements, implying that the dissemination of *dnd* systems depends mainly on HGT events^45^. This notion was in line with the results of the phylogenetic analysis of *dnd* genes and PT sequence contexts^19,26^. To address the hypothesis against the ecological paradox concerning the distribution of the *dnd* system, derivatives of the marine bacterium *S. piezotolerans* WP3 and filamentous phage SW1 were used as a model system in this study to investigate the influence of PT modification on the transcription and physiology of microbes. Owing to their versatile metabolic capabilities, members of the genus *Shewanella* inhabit diverse environments, including seawater, sediment, deep ocean, marine invertebrates, food, and occasionally clinical samples^46–49^. A previous survey indicated that only 3 *Shewanella* species harbour a *dnd* system, leaving the majority of *Shewanella* not modified by PT^19^. Filamentous phages from the *Inoviridae* family are pervasive in prokaryotes across Earth’s biomes^50,51^, and they have been identified in diverse environments and belong to the dominant virus groups in marine sediment^52–55^. Moreover, due to their unique characteristic of not lysing host cells even in the induced state, they are very suitable for exploring the influences after prophage activation^56^. Previously, *dnd* genes have been identified in several marine bacteria and oceanic metagenomes, and PT modification has been detected in DNA samples from the Oregon coast and the Sargasso Sea at varying depths^26^. Therefore, the experiment conducted in this study simulated the HGT of a *dnd* system into a non-PT *Shewanella* bacterium, which probably occurs in natural marine environments.

As the most abundant biological entities on the planet, viruses, including bacteriophages, significantly influence the physiology, metabolism and life cycle of their microbial hosts^57–60^. Specifically, prophages are highly abundant in prokaryotic genomes^61,62^, and they could be considered as dangerous molecular time bombs due to their potential to be activated^63^. In this study, the significant negative correlation between the presence of prophages and a *dnd* system was illustrated (Fig. 3), and the activation of filamentous phages triggered by PT modification was experimentally confirmed. Noteworthily, *Caudovirales*, including *Siphoviridae*, *Myoviridae*, and *Podoviridae*, were predominant, accounting for 99.39% of all the taxonomically classified prophages in the present study (*n* = 17822) (Supplementary Fig. S21 and Data 2). Furthermore, phage-borne repressors or regulators were identified in the majority (59.68%) of all these prophage elements, suggesting that a similar PT-induced activation may be widespread among taxonomically diverse bacteriophages. To confirm the activation of other types of prophages triggered by PT modification, we reanalyzed previously reported transcriptomic data of *E. coli* K-12 strain JW3350^64^, which contains 9 different prophage elements in its genome (Supplementary Fig. S22). Among the 9 prophages, 6 contained a total of 14 genes whose transcription levels were significantly upregulated by PT modification. Notably, many of these upregulated genes encode proteins that have lethal effects on the bacterial host, such as holin, lysozyme, murein endopeptidase in DLP12 and Qin, cell death peptidase in e14, and toxin YeeV in CP4-44. Next, transformation assays were performed to verify whether the increasing expression of prophage genes due to PT modification would affect the transfer of plasmid DNA (Supplementary Fig. S22). As expected, the transformation frequency of the plasmid pSW2Dnd carrying the complete PT modification function was significantly lower than that of the control plasmids pSW2DndΔE and pSW2. To test the pervasiveness of this effect, we further conducted plasmid transfer assays in 2 *Shewanella* strains carrying multiple prophages (4 and 3 prophages in *S. oneidensis* MR-1 and *S. putrefaciens* W3-18-1, respectively), and similar results were obtained (Supplementary Fig. S23). These experimental evidences strongly suggest that the activation of the prophage gene and the subsequent detrimental consequence caused by PT modification probably led to HGT failure of the *dnd* system, thereby impeding its dissemination among prokaryotic genomes (Fig. 7).

Epigenetic modifications of DNA, which involve no alterations in the nucleic acid sequence, play important roles in cellular physiology^65^. The most well-studied DNA modifications are base methylations, including the forms of 6-methyladenosine (m6A), 4-methylcytosine (m4C), and 5-methylcytosine (m5C), which are actively involved in the regulation of the expression of various genes^66,67^. DNA methylase is presumably derived from the more ancient RNA methylase, which could be traceable to the last universal common ancestor (LUCA) of all life^68,69^. As a novel type of modification of the DNA backbone, PT modification is also speculated to participate in gene regulation^19^. Previously, although 184 genes were identified as being differentially transcribed in the *dndBCDE* deletion mutant of *S. enterica*, subsequent experiments indicated that these changes were due to the SOS response resulting from the activity of DndFGH^70^. The global transcriptional impact of PT modification was further investigated in *P. fluorescens* pf0-1, which harbours *dndBCDE* but lacks the cognate *dndFGH*^19^. However, only 10 DEGs were observed between the wild-type strain and the *dndBCDE* deletion mutant, and none of the promoter regions of these DEGs contained PT sites^19^. Nevertheless, an in vitro transcription assay indicated that PT modification disturbed gene transcription (4 genes out of the 10 tested genes)^19^. Recently, *dndB* gene expression was shown to be regulated by PT modification in *S. lividans*^22^. Generally, although the in vivo evidence is not convincing, these results strongly suggest that PT modification is involved in epigenetic regulation. The key relevant step forward is to verify whether and how PT modification is capable of regulating the transcription of non-*dnd* genes in vivo. In this study, the transcription of various functional genes was significantly altered in the PT-modified strain, thus providing important evidence further supporting the epigenetic regulation of PT modification.

Interestingly, it seemed that PT modifications are not directly correlated with the transcriptional changes of genes based on coupling analysis of modification and transcriptomic data. This phenomenon has been previously noted in *P. fluorescens* Pf0-1, and reasonable explanations were given accordingly^19^. Regarding this special feature of PT-dependent epigenetic regulation, we propose that an in-depth analysis, especially at the single-cell or single-molecule level, will facilitate determining the underlying mechanism in the future. Despite this unresolved problem, we proved in this study that PT modification can alter the binding affinity between transcriptional regulators and their cognate operator DNA, thereby causing changes in gene transcription (Fig. 5). The underlying mechanism at the molecular level can be further explained by the results of a previous theoretical study. Specifically, Rp-phosphorothioation (Rp-PT), which is the biologically produced isomer of the PT modification, substantially destabilized B-type DNA, enhanced the rigidity of the DNA backbone, and differentiated backbone stability as an interaction with base steps^71^. Therefore, we assumed that the interactions between PT and bases can alter the conformation of DNA and then influence the DNA-protein binding affinity.

As a multifunctional epigenetic system, the *dnd* system plays roles in defence against foreign DNA, the balance of intracellular redox homeostasis, antioxidation, and virus resistance^9^. Despite these advantages, deficiencies in PT modification have also been reported. For example, the PT modification of DNA leads to lethal genomic instability under hypochlorous acid stress^72^. Moreover, the antioxidant ability of PT modification is very weak; it could restore only approximately 50% of the antioxidant ability in the *E. coli* MG1655 strain with the loss of catalase and peroxidase^16^. Compared with that of other, more efficient antioxidant systems, such as those of catalase, SOD and SOR^73^, the survival advantage of the *dnd* system conferred to the host is probably not substantial, thereby reducing the possibility that it is retained by the microbial population after HGT. Correspondingly, the introduction of the *dnd* gene cluster did not promote the growth of *S. piezotolerans* WP3 cells at its optimal temperature^16^. In contrast, a marked competitive disadvantage was noticed (Fig. 6b). To further support this notion, the *dndBCDE* gene cluster was transformed into *E. coli* MG1655, which harbor an intact antioxidant system. Unlike the situation in the Hpx^−^ strain, which is a catalase/peroxidase-disrupting strain^16^, the horizontally transferred PT system did not afford *E. coli* MG1655 growth advantages under multiple stress conditions (Supplementary Fig. S24). In accordance with this viewpoint, *dnd* systems were found to be preferentially maintained in some pathogens, such *Mycobacterium abscessus* (~50% of clinical isolates of *M. abscessus* exhibit the PT phenotype) and *Clostridium difficile*, both of which lack catalase and SOD^74,75^, to compensate for their antioxidative activity.

In this study, an evolutionary scenario of the *dnd* system was proposed, and the underlying mechanism of restricted HGT was experimentally explored (Fig. 7). Our findings not only explain the paradox concerning the patchy distribution of *dnd* systems but also deepen our understanding of the relationship between the origin and dissemination of novel microbial functional traits and major events (especially the GOE) on Earth during long-term evolutionary history.

## Methods

### Identification of *dnd* genes and prophages in prokaryotes

In total, the sequence data of 22,280 and 388 bacterial and archaeal genomes were retrieved from the RefSeq genome database (Release 202) in December 2020. To avoid the effect of low genomic quality on the analysis of the distribution of the *dnd* system, only the “assembly level” of “complete genome” or “chromosome” was selected. The Dnd protein sequences in the Kyoto Encyclopedia of Genes and Genomes (KEGG) Orthology (KO) database of the KEGG database^76^, including DndA/IscS (K04487), DndB (K19169), DndC (K19170), DndD (K19171), DndE (K19172), DndF (K19173), DndG (K19174), and DndH (K19175), were retrieved and used to construct a reference protein database. Dnd proteins encoded by prokaryotic genomes were identified by BLASTp using DIAMOND (v0.9.25.126)^77^ against the reference database, with a cut-off value of e < 10^−10^, a coverage ≥50% and a parameter of “—more-sensitive”, as used in a previous study^19^. The *dndCD*, *dndBCDE* and *dndFGH* gene clusters were considered to be present when all *dnd* genes were adjacent. Additionally, the occurrence of *dndFGH* in 30 ORFs upstream or downstream of *dndCD/dndBCDE* was a requirement, as previously described^19^. The prophages in prokaryotic genomes were identified and annotated by VIBRANT (v1.2.1), with the default parameters^78^. For the taxonomic classification of prophages, a majority-rules approach was used to assign viral taxonomy as previously described^79^. Briefly, all proteins from prophages were subjected to BLASTp alignment against RefSeq virus, and a prophage was considered to belong to a viral family if ≥50% of the proteins were assigned to that family with a bitscore ≥50.

### Phylogenetic analysis

To construct a phylogenetic tree of DndD, all DndD protein sequences were first identified by BLASTp (E-value cut off of 10^−20^ and query coverage of 75%) and then downloaded from the NCBI database, aligned by the MAFFT algorithm (v7.313)^80^, and filtered with trimAl (v1.2)^81^; a phylogenetic tree was then constructed using RAxML (v8.0)^82^ and the PROTGAMMAAUTO model, with 1,000 bootstraps; a phylogenetic tree of DndC was constructed with the same procedure. For the reference phylogenetic tree of prokaryotes, 120 and 122 concentrated conserved proteins were identified and aligned by GTDB-Tk (v1.3.0)^83^ from the 771 bacterial and 96 archaeal genomes, respectively. The tree was constructed by FastTree 2 (v2.1.10)^84^ based on the maximum-likelihood algorithm and visualized by MEGA X (v10.2.2)^85^. For clarity, only the bacterial orders and archaeal phyla with ≥30 high-quality genomes were included in the phylogenetic tree. For the phylogenomic tree of Cyanobacteria, a collection of sequences of cyanobacterial genomes was downloaded from the NCBI database. The tree was constructed via GTDB-Tk^83^ “classify_wf” and visualized by iTOL (v4)^86^; Sericytochromatia was set as the root lineage^44^. The phylogenetic tree of Cyanobacteriales DndD and the phylogenomic tree of Cyanobacteriales were constructed according to similar procedures.

### Correlation analyses between prophages and *dnd* genes/gene clusters

For effective statistical analyses^87^, only the genera and species with more than 100 and 30 genomes of high quality, respectively, were included for correlation analyses. Among these genera and species, the association between the occurrence of prophages and each *dnd* gene/gene cluster was measured with the Pearson correlation coefficient via the Python function “Pearsonr” from SciPy (v1.0)^88^ and linear regression analyses in R (v3.5.3)^89^. Within each genus and species, the association between the abundance of prophages and each *dnd* gene/gene cluster was measured with Kendall’s τ coefficient and *P*-values via the Python function “kendalltau” from SciPy^88^. Kendall’s τ coefficient was chosen for this because the values included a large number of “0” and “1” and varied on a low degree, which were not considered continuous normal data for the calculation of linear correlations but instead were considered ordinal categorical variables for the calculation of rank correlations^90^. To facilitate statistical analysis, only the genus and species with ≥30 genomes carrying at least 1 prophage or *dnd* gene/cluster were included.

### Bacterial strains and culture conditions

All bacterial strains and plasmids used in this study are listed in Supplementary Table S5. The *Shewanella* strains were cultured in modified 2216E marine media (2216E) (5 g/l tryptone, 1 g/l yeast extract, 0.1 g/l FePO_4_, 34 g/l NaCl) with shaking at 220 rpm at different temperatures. *E. coli* strains were incubated in lysogeny broth (LB) media (10 g/l tryptone, 5 g/l yeast extract, 10 g/l NaCl) supplemented with 50 μg/ml DL-α, ε-diaminopimelic acid (DAP) at 37 °C. For solid media, agar-A (Bio Basic Inc., Ontario, Canada) was added at 1.5% (w/v). The antibiotic chloramphenicol (Cm) (Sigma, St. Louis, USA) was added to the media at final concentrations of 25 μg/ml and 12.5 μg/ml for *E. coli* and *Shewanella*, respectively, when needed. The growth of the *Shewanella* and *E. coli* strains was determined using turbidity measurements at 600 nm in 2216E and LB media, respectively.

### Construction of PT modification strains

DNA fragments coding for the *dndA* and *dndBCD* genes were amplified via PCR using plasmids pJTU3619 and pJTU3529, respectively, as templates. The *dndA* products were cloned between *Xho*I and *Kpn*I into the corresponding sites of pSW2. The *dndBCD* fragments were then cloned between *Pst*I and *Kpn*I into the corresponding sites, yielding pSW2DndΔE. Vectors harbouring the *fpsR* deletion were constructed using the vectors pSW2Dnd and pSW2DndΔE. Briefly, two opposing primers targeting the *fpsR* gene were used to amplify the whole sequence of the two vectors, except for the coding region of *fpsR*. The PCR products were subsequently digested with *Apa*I and then self-ligated, yielding pSW2DndΔR and pSW2DndΔEΔR, respectively. For the construction of vectors with PT site mutations, fragments of *dndABCDE*, *dndABCD*, and pSW2 were amplified via PCR. The intergenic region between *fpsA-fpsR*, which harboured PT site mutations, was synthesized (Biosune, Shanghai, China) and then fused to *dndABCDE*/*dndABCD* and pSW2 fragments with a ClonExpress cloning kit (Vazyme, Nanjing, China), yielding pSW2Dnd-IG and pSW2DndΔE-IG, respectively. The vector constructs were transformed into WM3064, a DAP auxotrophic strain. The transformants were subsequently confirmed via enzyme digestion and DNA sequencing. The vectors were then introduced into WP3 strains by two-parent conjugation. The transconjugant was selected by Cm resistance and was verified via PCR. For construction of PT-modified *E. coli* strains, pJTU3619 and pJTU3529 plasmids were introduced into MG1655 cells by calcium chloride transformation. All the vectors were confirmed by DNA sequencing.

### Determination of PT modification in plasmid and genomic DNA

PT modification in plasmid DNA was detected by a peracetic acid (PAA) cleavage assay as previously described^15^. Briefly, the plasmid was linearized using *Xho*I or *EcoR*I according to the manufacturer’s instructions. The linearized plasmid DNA was dissolved in 1× TAE to a concentration of 300 ng/ml. A total amount of 30 ng/ml DNA was incubated in PAA–TAE, which was made by mixing 1% 1 M stock PAA with TAE buffer (40 mM Tris base adjusted to pH 7.5 using acetic acid and 0.8 mM ethylenediaminetetraacetic acid (EDTA)) for 20 min. DNA was precipitated using propanol and then resuspended in TAE buffer for agarose gel electrophoresis analysis. PT modifications in pSW2Dnd and WP3NR/Dnd were quantified by LC-coupled, time-of-flight mass spectrometry as previously described^26^. In brief, the DNA was digested using nuclease P1 and alkaline phosphatase. The digestion mixture containing PT dinucleotides was resolved on a Poroshell 120 SB-AQ column (Agilent Co., CA, USA). The high-performance LC (HPLC) column was then coupled to an Agilent 6410 Triple Quad LC–MS spectrometer (Agilent Co., CA, USA) with an electrospray ionization source in positive mode. Multiple reaction monitoring modes were used for the detection of product ions derived from the precursor ions. The instrument parameters, including precursor ion m/z, product ion m/z, fragmentor voltage and collision energy, were the same as previously described^26^.

### RNA isolation and RT-qPCR

The *S. piezotolerans* WP3 strains were inoculated into 2216E media, after which the culture was collected and immersed in liquid nitrogen immediately when the cells reached exponential phase. Total RNA was isolated with TRI reagent-RNA isolation kit (Molecular research center, Cincinnati, USA). The RNA samples were treated with DNase I at 37 °C for 1 h to remove DNA contamination. The purified RNA were reverse transcribed to cDNA by RevertAid First Strand cDNA Synthesis Kit (Fermentas, Maryland, USA). The primer pairs used to amplify the selected genes for RT-qPCR were designed using Primer Express software (v3.0.1) (Applied Biosystems, CA, USA). PCR cycling was conducted using 7500 System SDS software (v2.0.6) (Applied Biosystems) in 20 μl reaction mixtures that included 1× SYBR Green I Universal PCR Master Mix (Applied Biosystems), 0.5 μM each primer, and 1 μl cDNA template^91,92^.

### Phage DNA copy number determination

The copy numbers of pSW2 RF DNA and ssDNA were quantified as previously described^93^. In brief, a pair of primers, SW1RFRTFor/SW1RFRTRev (Supplementary Table S6), were designed to quantify the copy number of RF DNA; the primer pair targeted the *attP* site of SW1, and RF DNA was used as the amplification template. Total DNA was used as a template for the first round of qPCR. The copy number equalled the number of RF DNA plus ssDNA combined. The template for the second round of qPCR was the total DNA treated with S1 nuclease (Thermo Fisher Scientific, MA, USA). After the ssDNA was removed from the total DNA, the quantification result was the copy number of RF DNA. Finally, the copy number of ssDNA was calculated from the two rounds of qPCR.

### Nuclease activity assays

To assess intracellular nuclease activity, crude enzyme extracts of WP3NR strains were incubated together with DNA substrate. In particular, the WP3NR strains from cultures at the mid-logarithmic phase (OD_600_ = 1.0) were pelleted by centrifugation. The harvested cell pellets were resuspended in lysis buffer [500 mM NaCl, 10% glycerol (w/v), 20 mM Tris–HCl (pH 8.0)] and sonicated on ice. The cell lysates were subsequently centrifuged at 10,000 × *g* for 20 min at 4 °C, after which the supernatants were obtained. Nine microliters of the crude enzyme extracts were incubated together with approximately 100 ng of purified DNA (PCR-amplified 16 S rRNA gene and genomic DNA of *S. piezotolerans* WP3) in reaction buffer (Takara, Dalian, China). The reaction mixtures were incubated at 20 °C for 1 h and then separated on a 1% agarose gel. The DNA was then stained with GelRed (Biotium, Hayward, USA) and visualized by a gel imaging system (Tanon, Shanghai, China).

### Expression and purification of FpsR

FpsR was expressed and purified as previously described^33^. Briefly, *E. coli* strain C41 (DE3) containing the FpsR expression vector was grown in 1 l of LB broth with 50 μg/ml kanamycin at 37 °C for 3 h. FpsR expression was induced by the addition of 0.5 mM isopropyl-β-D-thiogalactopyranoside (IPTG) when the OD_600_ reached 1.0, and the culture was then incubated at 20 °C overnight. The cells were collected by centrifugation, resuspended in binding buffer (500 mM NaCl and 20 mM imidazole, 20 mM Tris-HCl, pH 8.0) and sonicated on ice. The cell extract was clarified by centrifugation at 10,000 × *g* for 20 min at 4 °C. Ni Sepharose High Performance (GE Healthcare, WI, USA) resin was used to purify the His-tagged FpsR according to the manufacturer’s instructions. The protein was eluted in elution buffer (500 mM NaCl and 500 mM imidazole, 20 mM Tris-HCl, pH 8.0). Imidazole was removed using HiTrap Desalting columns (GE Healthcare, WI, USA) according to the manufacturer’s instructions. The purified FpsR was stored at 4 °C, and its concentration was determined by the Bradford method using bovine serum albumin (BSA) as a standard. The purity of FpsR was confirmed by SDS-PAGE (15%) with visualization using Coomassie Brilliant Blue R-250.

### In vitro transcription

In vitro transcription was performed as previously described^19^, with slight modifications. Briefly, PT-modified oligonucleotides (ssDNA) were chemically synthesized (Sangon Biotech, Shanghai, China), and annealed to obtain PT-modified dsDNA; non–PT-modified DNA templates were generated by PCR amplification. Both DNA templates were purified using a GenElute PCR Clean-Up Kit (Sigma, St. Louis, USA) and then quantified by using an Invitrogen Qubit dsDNA High-Sensitivity (HS) Assay Kit (Thermo Fisher Scientific, MA, USA). Afterwards, 100 ng of DNA template was transcribed in a 20-μl reaction mixture that included 2 units of E. coli RNA polymerase, holoenzyme (New England Biolabs, MA, USA), 4 μl of 5× *E. coli* RNA polymerase reaction buffer, 0.5 mM NTP Mix (Thermo Fisher Scientific, MA, USA), and 20 units of RNase inhibitor (Solarbio, Beijing, China). In vitro transcription was conducted at 37 °C for 12 h, after which the temperature was increased to 85 °C for 10 min to stop the reaction. The RNA product was quantified by using an Invitrogen Qubit RNA HS Assay (Thermo Fisher Scientific, MA, USA).

### SPR measurements

The DNA probe used for SPR was a 50-bp fragment that included the FpsR binding site and PT site (Supplementary Table S6). Oligonucleotides (with and without PT modification) were chemically synthesized (Biosune, Shanghai, China) and purified with a Cycle Pure Kit (Omega Bio-Tek, Norcross, USA). The SPR measurements were performed via a Biacore 8 K instrument (GE Healthcare, WI, USA), as previously described^30^, with slight modifications. Briefly, 10 nM biotinylated DNA was captured on the surface of a streptavidin sensor chip (Cytiva, MA, USA) at a flow rate of 30 μl/min for 120 s in phosphate-buffered saline (PBS) consisting of 0.05% (v/v) Tween-20 (pH 7.4). A series of concentrations of FpsR proteins were injected into the flow system and analyzed. All binding analyses were performed in PBS consisting of 0.05% (v/v) Tween-20 (pH 7.4), at both 4 °C and 20 °C. Both the association and dissociation times were set to 120 s. After dissociation, the chip surface was regenerated with 0.5% SDS (w/v) for 30 s and stabilized for 120 s. Prior to analysis, double reference subtractions were performed to eliminate bulk refractive index changes, injection noise, and data drift. The binding affinity was determined by global fitting to a Langmuir 1:1 binding model within the Biacore insight evaluation software (v1.0) (GE Healthcare, WI, USA)^91,92^.

### Genomic mapping of PT modification by SMRT sequencing

SMRT sequencing and PT modification detection were performed as previously described^19,20,29^. In brief, the genomic DNA of WP3 was fragmented to an average size of 20 kb using g-TUBEs (Covaris, Woburn, MA, USA). The fragmented DNA was end repaired and ligated to hairpin adaptors. SMRT sequencing was carried out on a PacBio RS II instrument (Pacific Biosciences, Menlo Park, CA, USA). A total of 886,401,450 bp of sequencing data were produced, with an average read length of 5,897 bp. The genome was then assembled using HGAP 3.0^94^ with the default parameters in SMRT Analysis Suite (v1.3) (Pacific Biosciences, Menlo Park, CA, USA). Base modification analysis was performed using the base modification detection workflow of SMRT Analysis v1.3, and the PT modifications in the whole genome were identified according to calculations of the interpulse duration (IPD) ratio as previously described^29^.

### Transcriptomic analysis

Strand-specific transcriptome sequencing was performed at Magigene Biotechnology Co., Ltd. (Guangdong, China). Briefly, rRNA was removed using an Epicentre Ribo-Zero rRNA Removal Kit (Epicentre, Madison, WI, USA), and a cDNA library was prepared with a NEBNext Ultra II Directional RNA Library Prep Kit for Illumina (NEB, Ipswich, MA, USA) according to the manufacturer’s instructions. The initial quantification of the library was carried out using a Qubit Fluorometer (Life Technologies, Carlsbad, CA, USA), and the insertion fragment size of the library was determined with an Agilent 2100 Bioanalyzer (Agilent Technologies, Palo Alto, CA, USA). The effective concentration of the library was quantified accurately via qPCR (effective concentration >2 nM). The different libraries were pooled together in a flow cell according to the effective concentration and the target offline data volume. After clustering, the Illumina HiSeq sequencing platform (Illumina, San Diego, USA) was used for paired-end sequencing. The raw data were filtered and evaluated by fastp software (v0.19.7)^95^, after which the clean reads were mapped to the *S. piezotolerans* WP3 genome (NC_011566.1) by HISAT software (v2.1.0)^96^. RSEM (v1.3.1)^97^ was used to calculate the read counts per sample, and the sequencing results were evaluated in terms of quality, alignment, saturation, and distribution of reads on the reference genome by DEGseq (v1.36.0)^98^. Gene expression was calculated on the basis of the number of reads mapped to each gene using the fragments per kilobase per million mapped reads (FPKM) method^99^ and analyzed by edgeR (v3.20.2)^100^. The DEGs were identified according to the following standards: a false discovery rate (FDR) < 0.05 and an FPKM fold change ≥ 2 between two samples.

### Competition assays

Cultures of WP3NR/Dnd and WP3NR/DndΔE were grown independently to the stationary phase in modified 2216E media to an OD_600_ of 3.5. A total of 5 ml of each culture was mixed together and taken as the T0 sample, and 100 μl of the same mixture was inoculated into 9.9 ml of fresh 2216E media supplemented with Cm at a final concentration of 12.5 μg/ml. After incubation for 24 h, 100 μl of the competing cells was inoculated into 9.9 ml of fresh 2216E media, and the rest was taken as the T1 sample. The experiment was repeated the next day, and the sample was collected as T2. In total, the procedure was performed for 5 consecutive days^101^. All the samples were serially diluted with fresh 2216E media, and 100-μl aliquots of appropriately diluted samples were plated onto 2216E plates. A total of 100 colonies from plates containing 100–400 colonies were randomly picked and subjected to colony PCR in conjunction with the primers listed in Supplementary Table S6. The relative fitness (*W*)^102^ was calculated by the ratio of the number of doubling of WP3NR/Dnd and WP3NR/DndΔE.

### Plasmid transfer assays

Three plasmids (pSW2Dnd, pSW2DndΔE and pSW2) were introduced into *E. coli* BW25113 and JW3350 cells by calcium chloride transformation. The number of transformants was determined by counting colonies on selective agar medium and was verified via PCR. The transformation frequency was calculated as the number of transformants per μg plasmid DNA. For conjugal transfer, these 3 plasmids were introduced into *S. oneidensis* MR-1 and *S. putrefaciens* W3-18-1 strains by two-parent conjugation between them and the plasmid harbouring *E. coli* WM3064 strains. The transconjugant was selected by Cm resistance and was verified via colony PCR. The conjugation frequency was calculated as the number of transconjugants per number of donors.

### Reporting summary

Further information on research design is available in the Nature Research Reporting Summary linked to this article.

## Supplementary information

Supplementary Information

Description of Additional Supplementary Files

Dataset 1

Dataset 2

Dataset 3

Dataset 4

Reporting Summary

## Data Availability

All bacterial and archaeal assembled genomes (*n* = 22,668) used in this study were collected from the NCBI RefSeq database (Release 202, https://www.ncbi.nlm.nih.gov/refseq/). The Dnd protein sequences were retrieved from the KEGG Orthology (KO) database (https://www.genome.jp/kegg/ko.html). All the detailed information of the identified *dnd* genes, gene clusters and prophages are described and available publicly in Supplementary Data 1 and 2. The epigenomic sequencing data of *S. piezotolerans* WP3NR/Dnd generated using the PacBio RSII platform are available in Figshare [https://figshare.com/articles/dataset/SMRT_sequencing_data_of_Shewanella_piezotolerans_WP3NR_Dnd/16809943]. The transcriptomic data from the current study have been deposited in the NCBI SRA under project ID PRJNA565632. Source data are provided with this paper.

## Source data

Source data
