# Supplementary material for: The origin and impeded dissemination of the DNA phosphorothioation system in prokaryotes
Source: Nat Commun. 2021 Nov 4;12:6382. doi: 10.1038/s41467-021-26636-7 (PMC8569181; doi:10.1038/s41467-021-26636-7)
Supplement: Supplementary file 1 — Supplementary Information [file 41467_2021_26636_MOESM1_ESM.pdf]

Supplementary Information

The origin and impeded dissemination of the DNA phosphorothioation system in prokaryotes

Authors: Huahua Jian[1, 2†], Guanpeng Xu[1†], Yi Yi[1†], Yali Hao[1], Yinzhao Wang[1], Lei Xiong[3], Siyuan Wang[1], Shunzhang Liu[1], Canxing Meng[1], Jiahua Wang[1], Yue Zhang[1], Chao Chen[3], Xiaoyuan Feng[1, 4], Haiwei Luo[4], Hao Zhang[4], Xingguo Zhang[5], Lianrong Wang[3], Zhijun Wang[1], Zixin Deng[1], Xiang Xiao[1, 2*]

*Correspondence. E-mail: zjxiao2018@sjtu.edu.cn

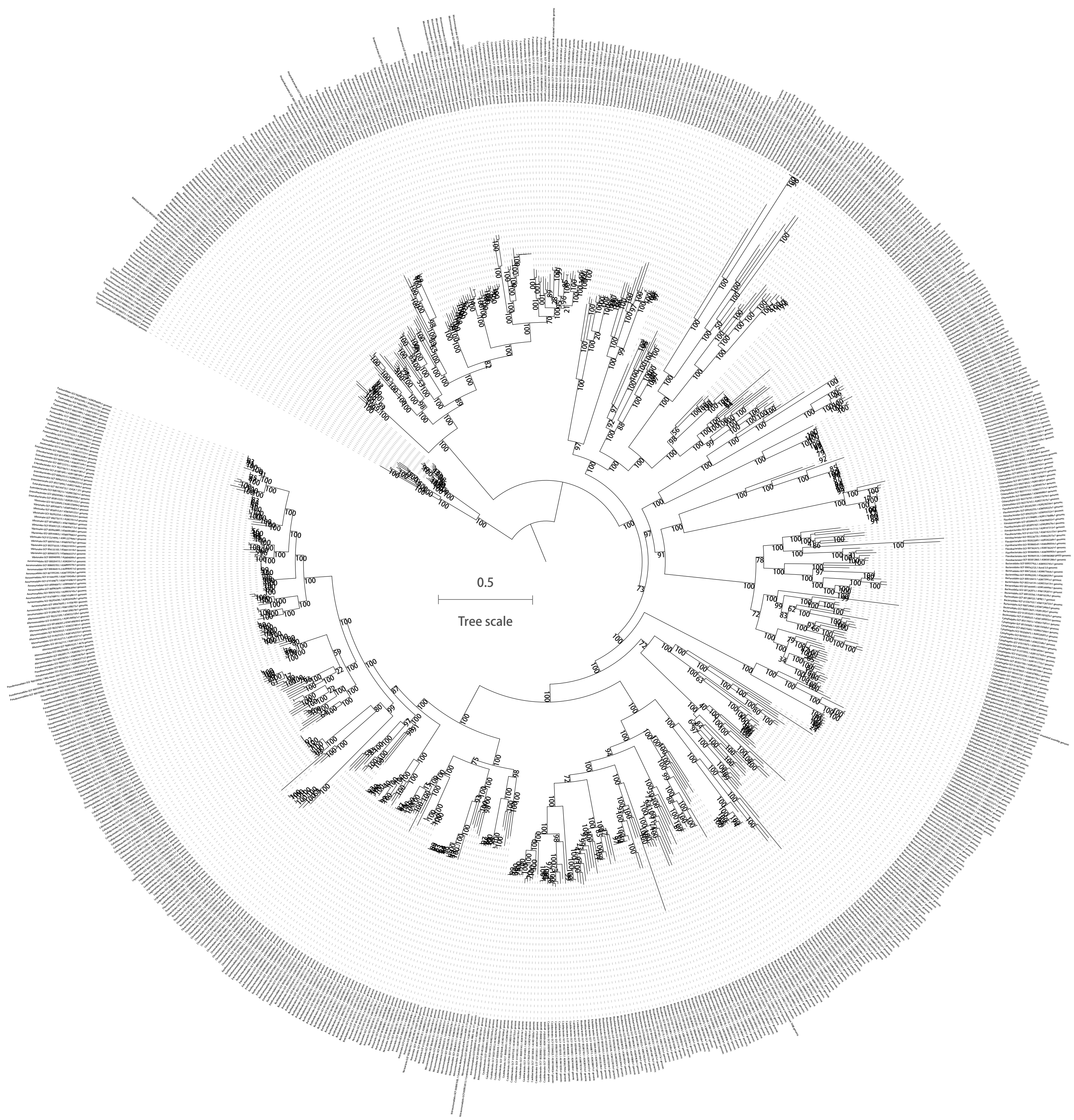
0.5
Tree scale

Fig. S1. Reference phylogenetic tree of major lineages of bacteria. A total of 120 conserved proteins from 771 bacterial genomes were identified, concatenated and aligned by GTDB-Tk[1]. The tree was reconstructed using FastTree v2.1.9[2] based on the maximum-likelihood algorithm and visualized by iTOL[3]. Sericytochromatia was set as the root lineage. Bootstrap support estimated from 1,000 replicates is listed below or above each branch. Only the bacterial orders with ≥ 30 high-quality genomes are shown.

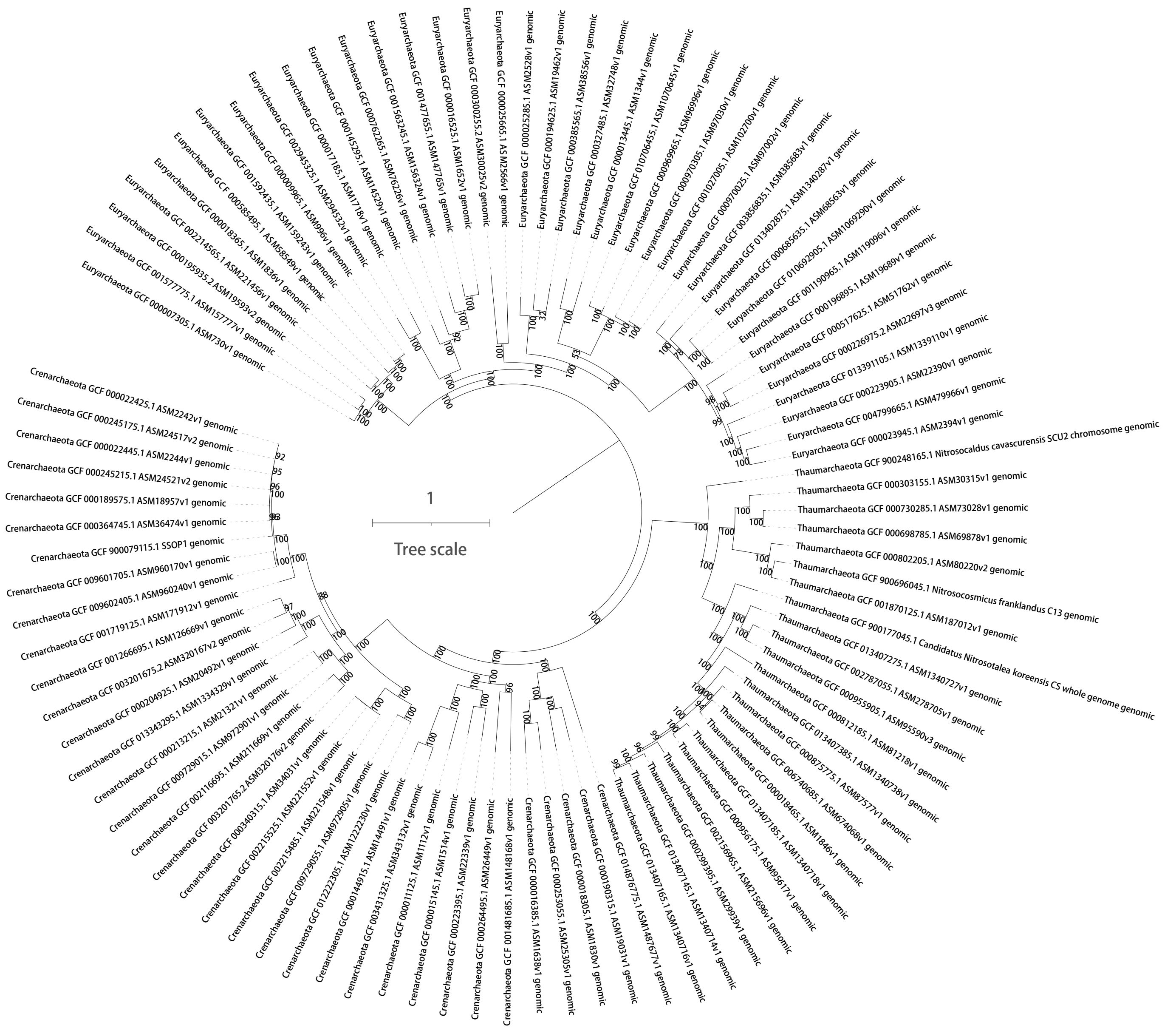
Tree scale
1
Euryarchaeota GCF 000007305.1 ASM730v1 genomic
Euryarchaeota GCF 001577775.1 ASM157777v1 genomic
Euryarchaeota GCF 000195935.2 ASM19593v2 genomic
Euryarchaeota GCF 002214565.1 ASM221456v1 genomic
Euryarchaeota GCF 000018365.1 ASM1836v1 genomic
Euryarchaeota GCF 000585495.1 ASM58549v1 genomic
Euryarchaeota GCF 001592435.1 ASM159243v1 genomic
Euryarchaeota GCF 000009965.1 ASM996v1 genomic
Euryarchaeota GCF 002945325.1 ASM294532v1 genomic
Euryarchaeota GCF 000017185.1 ASM1718v1 genomic
Euryarchaeota GCF 000145295.1 ASM14529v1 genomic
Euryarchaeota GCF 000762265.1 ASM76226v1 genomic
Euryarchaeota GCF 001563245.1 ASM156324v1 genomic
Euryarchaeota GCF 001477655.1 ASM147765v1 genomic
Euryarchaeota GCF 000016525.1 ASM1652v1 genomic
Euryarchaeota GCF 000300255.2 ASM30025v2 genomic
Euryarchaeota GCF 000025665.1 ASM2566v1 genomic
Euryarchaeota GCF 000025285.1 ASM2528v1 genomic
Euryarchaeota GCF 000194625.1 ASM19462v1 genomic
Euryarchaeota GCF 000385565.1 ASM38556v1 genomic
Euryarchaeota GCF 000327485.1 ASM32748v1 genomic
Euryarchaeota GCF 000013445.1 ASM1344v1 genomic
Euryarchaeota GCF 010706455.1 ASM1070645v1 genomic
Euryarchaeota GCF 000969965.1 ASM96996v1 genomic
Euryarchaeota GCF 000970305.1 ASM97030v1 genomic
Euryarchaeota GCF 001027005.1 ASM102700v1 genomic
Euryarchaeota GCF 000970025.1 ASM97002v1 genomic
Euryarchaeota GCF 003856835.1 ASM385683v1 genomic
Euryarchaeota GCF 013402875.1 ASM1340287v1 genomic
Euryarchaeota GCF 000685635.1 ASM68563v1 genomic
Euryarchaeota GCF 010692905.1 ASM1069290v1 genomic
Euryarchaeota GCF 001190965.1 ASM119096v1 genomic
Euryarchaeota GCF 000196895.1 ASM19689v1 genomic
Euryarchaeota GCF 000517625.1 ASM51762v1 genomic
Euryarchaeota GCF 000226975.2 ASM22697v3 genomic
Euryarchaeota GCF 013391105.1 ASM1339110v1 genomic
Euryarchaeota GCF 000223905.1 ASM22390v1 genomic
Euryarchaeota GCF 004799665.1 ASM479966v1 genomic
Euryarchaeota GCF 000023945.1 ASM2394v1 genomic
Thaumarchaeota GCF 900248165.1 Nitrosocaldus cavascurensis SCU2 chromosome genomic
Thaumarchaeota GCF 000303155.1 ASM30315v1 genomic
Thaumarchaeota GCF 000730285.1 ASM73028v1 genomic
Thaumarchaeota GCF 000698785.1 ASM69878v1 genomic
Thaumarchaeota GCF 000802205.1 ASM80220v2 genomic
Thaumarchaeota GCF 900696045.1 Nitrosocosmicus franklandus C13 genomic
Thaumarchaeota GCF 001870125.1 ASM187012v1 genomic
Thaumarchaeota GCF 900177045.1 Candidatus Nitrosotalea koreensis CS whole genome genomic
Thaumarchaeota GCF 013407275.1 ASM1340727v1 genomic
Thaumarchaeota GCF 002787055.1 ASM278705v1 genomic
Thaumarchaeota GCF 000955905.1 ASM95590v3 genomic
Thaumarchaeota GCF 000812185.1 ASM81218v1 genomic
Thaumarchaeota GCF 013407385.1 ASM1340738v1 genomic
Thaumarchaeota GCF 000875775.1 ASM87577v1 genomic
Thaumarchaeota GCF 006740685.1 ASM674068v1 genomic
Thaumarchaeota GCF 000018465.1 ASM1846v1 genomic
Thaumarchaeota GCF 013407185.1 ASM1340718v1 genomic
Thaumarchaeota GCF 000956175.1 ASM95617v1 genomic
Thaumarchaeota GCF 002156965.1 ASM215696v1 genomic
Thaumarchaeota GCF 000299395.1 ASM29939v1 genomic
Thaumarchaeota GCF 013407145.1 ASM1340714v1 genomic
Thaumarchaeota GCF 013407165.1 ASM1340716v1 genomic
Thaumarchaeota GCF 014876775.1 ASM1487677v1 genomic
Crenarchaeota GCF 000190315.1 ASM19031v1 genomic
Crenarchaeota GCF 000018305.1 ASM1830v1 genomic
Crenarchaeota GCF 000253055.1 ASM25305v1 genomic
Crenarchaeota GCF 000016385.1 ASM1638v1 genomic
Crenarchaeota GCF 001481685.1 ASM148168v1 genomic
Crenarchaeota GCF 000264495.1 ASM26449v1 genomic
Crenarchaeota GCF 000223395.1 ASM22339v1 genomic
Crenarchaeota GCF 000015145.1 ASM1514v1 genomic
Crenarchaeota GCF 000011125.1 ASM1112v1 genomic
Crenarchaeota GCF 003431325.1 ASM343132v1 genomic
Crenarchaeota GCF 000144915.1 ASM14491v1 genomic
Crenarchaeota GCF 012222305.1 ASM1222230v1 genomic
Crenarchaeota GCF 009729055.1 ASM972905v1 genomic
Crenarchaeota GCF 002215485.1 ASM221548v1 genomic
Crenarchaeota GCF 002215525.1 ASM221552v1 genomic
Crenarchaeota GCF 000340315.1 ASM34031v1 genomic
Crenarchaeota GCF 003201765.2 ASM320176v2 genomic
Crenarchaeota GCF 002116695.1 ASM211669v1 genomic
Crenarchaeota GCF 009729015.1 ASM972901v1 genomic
Crenarchaeota GCF 000213215.1 ASM21321v1 genomic
Crenarchaeota GCF 013343295.1 ASM1334329v1 genomic
Crenarchaeota GCF 000204925.1 ASM20492v1 genomic
Crenarchaeota GCF 003201675.2 ASM320167v2 genomic
Crenarchaeota GCF 001266695.1 ASM126669v1 genomic
Crenarchaeota GCF 001719125.1 ASM171912v1 genomic
Crenarchaeota GCF 009602405.1 ASM960240v1 genomic
Crenarchaeota GCF 009601705.1 ASM960170v1 genomic
Crenarchaeota GCF 900079115.1 SSOP1 genomic
Crenarchaeota GCF 000364745.1 ASM36474v1 genomic
Crenarchaeota GCF 000189575.1 ASM18957v1 genomic
Crenarchaeota GCF 000245215.1 ASM24521v2 genomic
Crenarchaeota GCF 000022445.1 ASM2244v1 genomic
Crenarchaeota GCF 000245175.1 ASM24517v2 genomic
Crenarchaeota GCF 000022425.1 ASM2242v1 genomic

Fig. S2. Reference phylogenetic trees of major lineages of archaea. A total of 122 conserved proteins from 97 archaeal genomes were identified, concatenated and aligned by GTDB-Tk[1]. The tree was reconstructed using FastTree v2.1.9[2] based on the maximum-likelihood algorithm and visualized by iTOL[3]. Bootstrap support estimated from 1,000 replicates is listed below or above each branch. Only the bacterial orders with ≥ 30 high-quality genomes are shown.

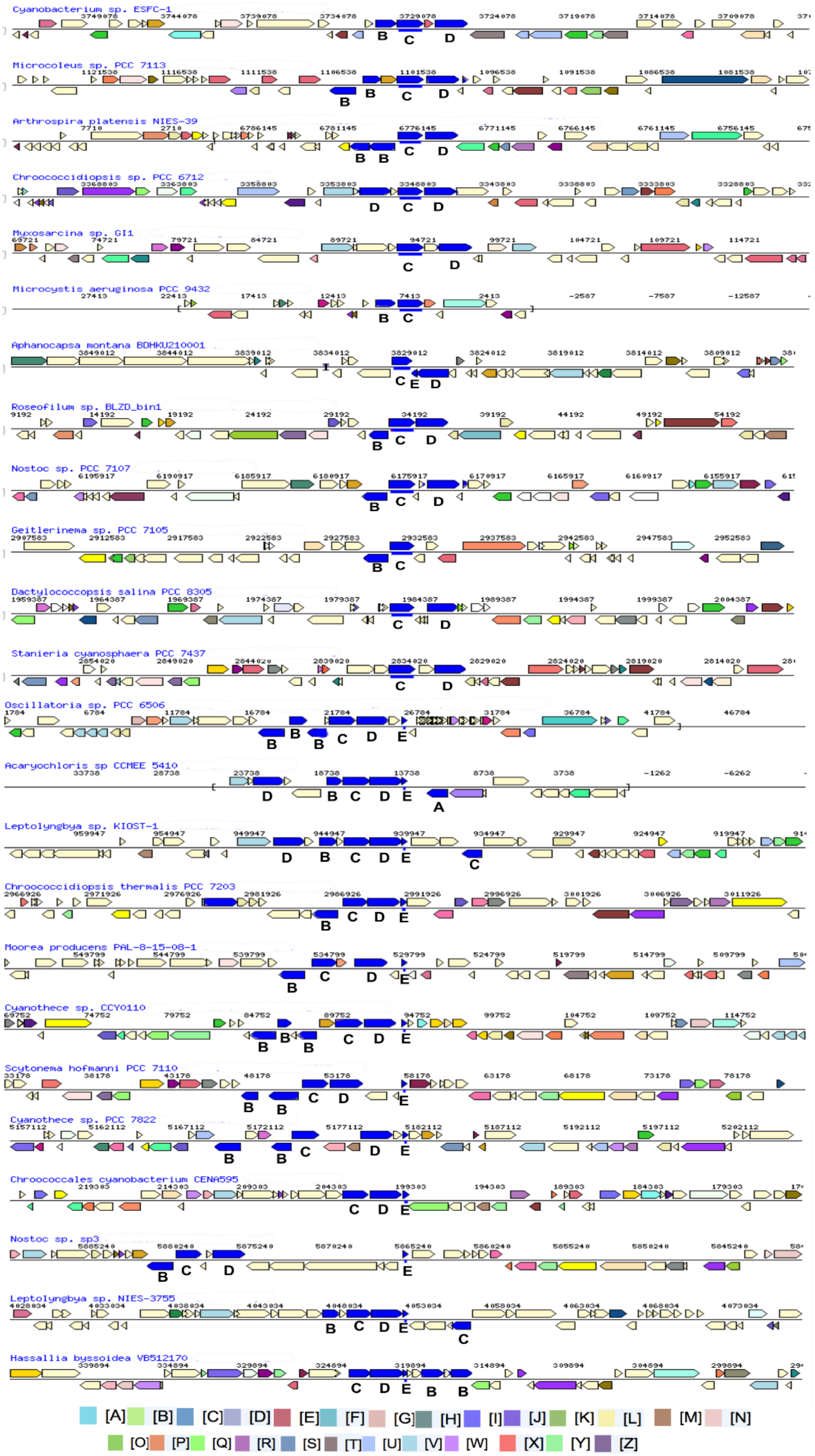

Cyanobacterium sp. ESFC-1
B C D
Microcoleus sp. PCC 7113
B B C D
Arthrospira platensis NIES-39
B B C D
Chroococcidiopsis sp. PCC 6712
D C D
Myxosarcina sp. GI1
C D
Microcystis aeruginosa PCC 9432
B C
Aphanocapsa montana BDHKU210001
C E D
Roseofilum sp. BLZD_bin1
B C D
Nostoc sp. PCC 7107
B C D
Geitlerinema sp. PCC 7105
B C
Dactylococcopsis salina PCC 8305
C D
Stanieria cyanosphaera PCC 7437
C D
Oscillatoria sp. PCC 6506
B B B C D E
Acaryochloris sp CCMEE 5410
D B C D E A
Leptolyngbya sp. KIOST-1
D B C D E C
Chroococcidiopsis thermalis PCC 7203
B C D E
Moorea producens PAL-8-15-08-1
B C D E
Cyanothece sp. CCY0110
B B B C D E
Scytonema hofmanni PCC 7110
B B C D E
Cyanothece sp. PCC 7822
B B C D E
Chroococcales cyanobacterium CENA595
C D E
Nostoc sp. sp3
B C D E
Leptolyngbya sp. NIES-3755
B C D E C
Hassallia byssoidea VB512170
C D E B B
[A] [B] [C] [D] [E] [F] [G] [H] [I] [J] [K] [L] [M] [N]
[O] [P] [Q] [R] [S] [T] [U] [V] [W] [X] [Y] [Z]

Fig. S3. The *dnd* gene cluster in cyanobacteria genomes. The *dnd* genes are indicated in blue. The *dndA*, *dndB*, *dndC*, *dndD* and *dndE* genes are indicated with A, B, C, D and E, respectively.

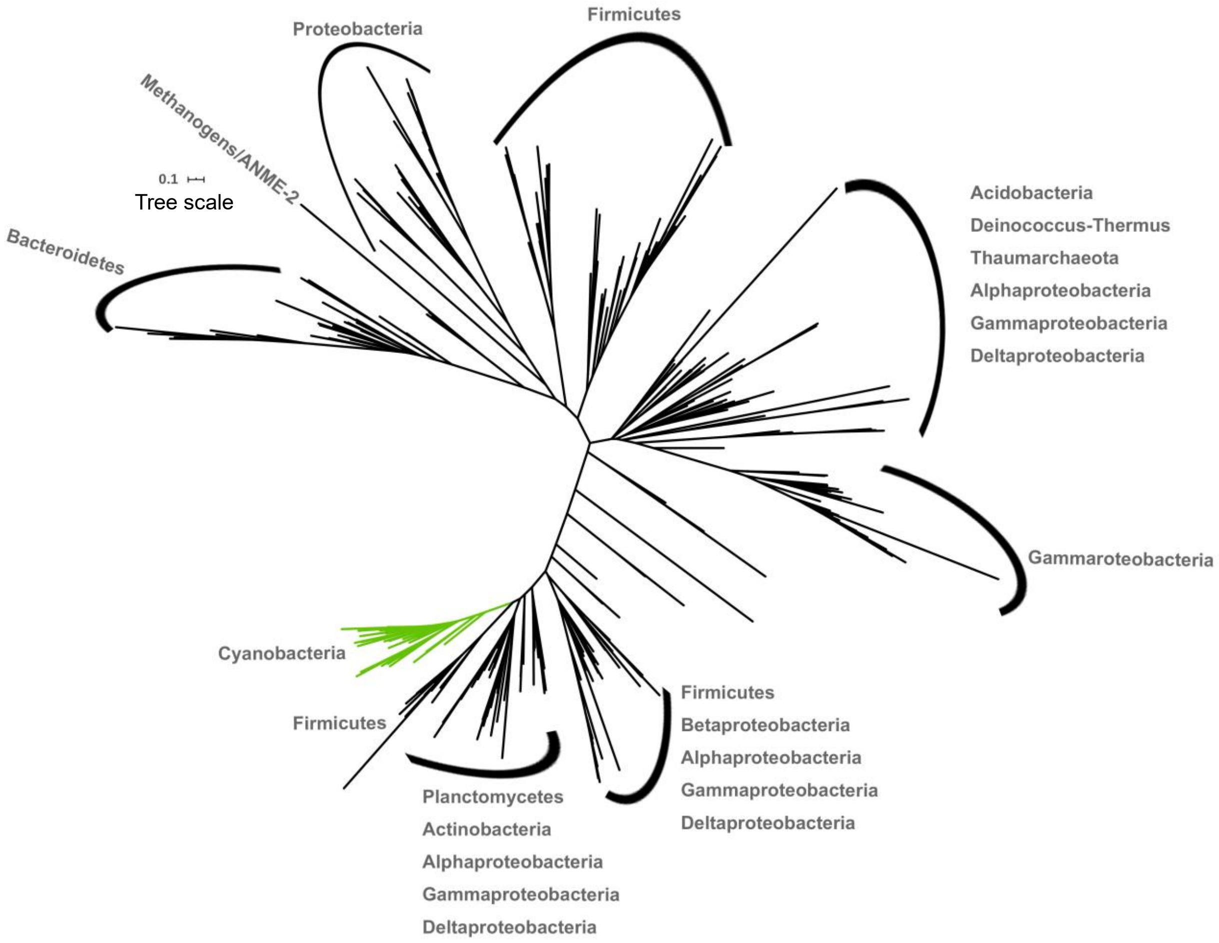

Fig. S4. Phylogenetic affiliations of DndC proteins. The phylogenetic tree was constructed based on the alignments of DndC that were generated using MAFFT and then filtered with trimAl, and the trees were built by the IQ-Tree method with model LG+C60+F+G and 1000 bootstrap replicates. The tree branches were classified into different bacterial or archaeal phyla or class groups. The green branch is Cyanobacteria, indicating an independent evolutionary path.

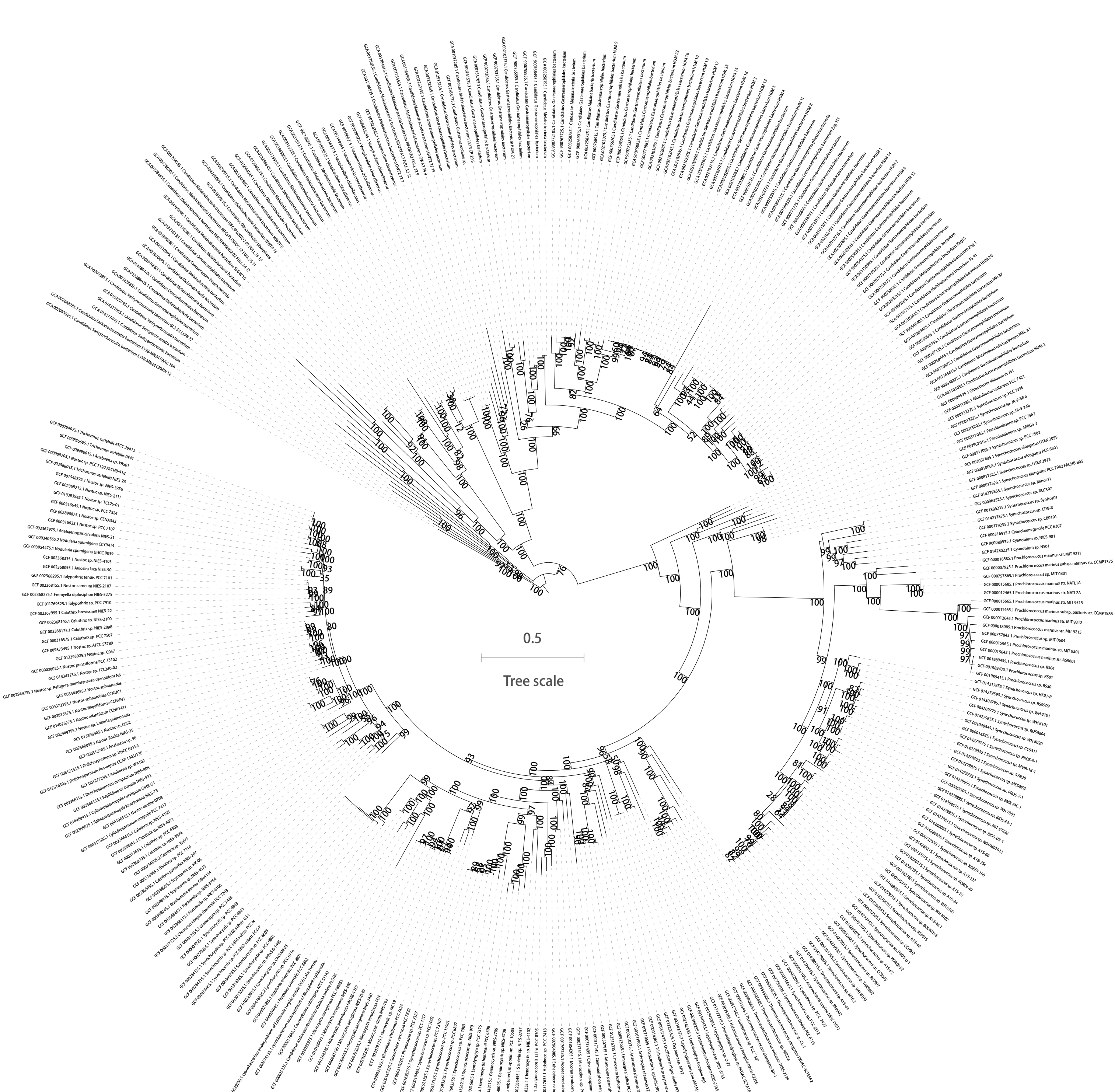
0.5
Tree scale

Fig. S5. Phylogenetic tree of Cyanobacteria. A total of 120 conserved proteins from Cyanobacteria genomes were identified, concatenated and aligned by GTDB-Tk[1]. The tree was reconstructed using FastTree v2.1.9[2] based on the maximum-likelihood algorithm and visualized by iTOL[3]. Sericytochromatia was set as the root lineage. Bootstrap support estimated from 1,000 replicates is listed below or above each branch.

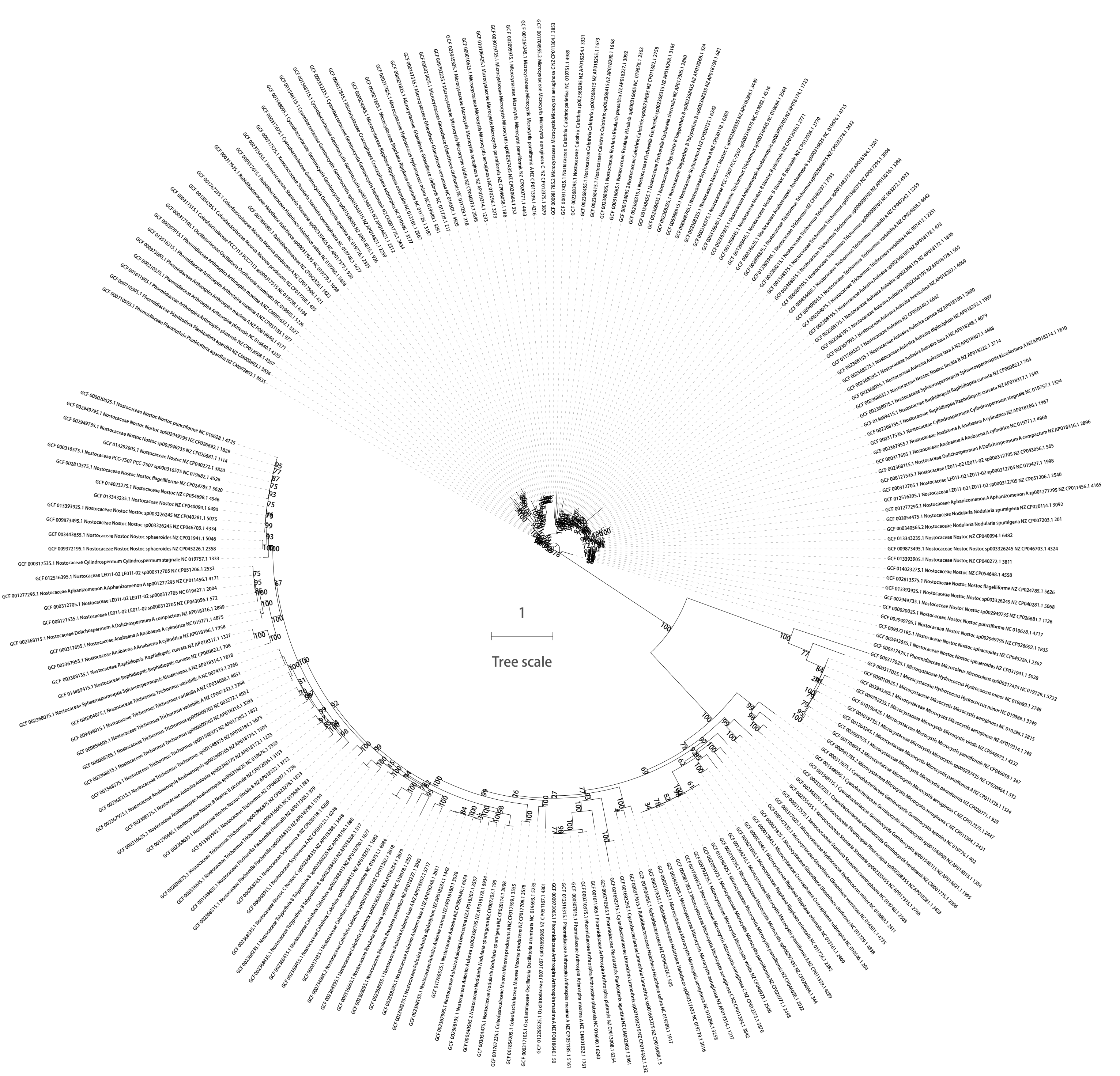
1
Tree scale

Fig. S6. Phylogeny of DndD in cyanobacteria. The tree was reconstructed using FastTree v2.1.9[2] based on the maximum-likelihood algorithm and visualized by iTOL[3]. Bootstrap support estimated from 1,000 replicates is listed below or above each branch.

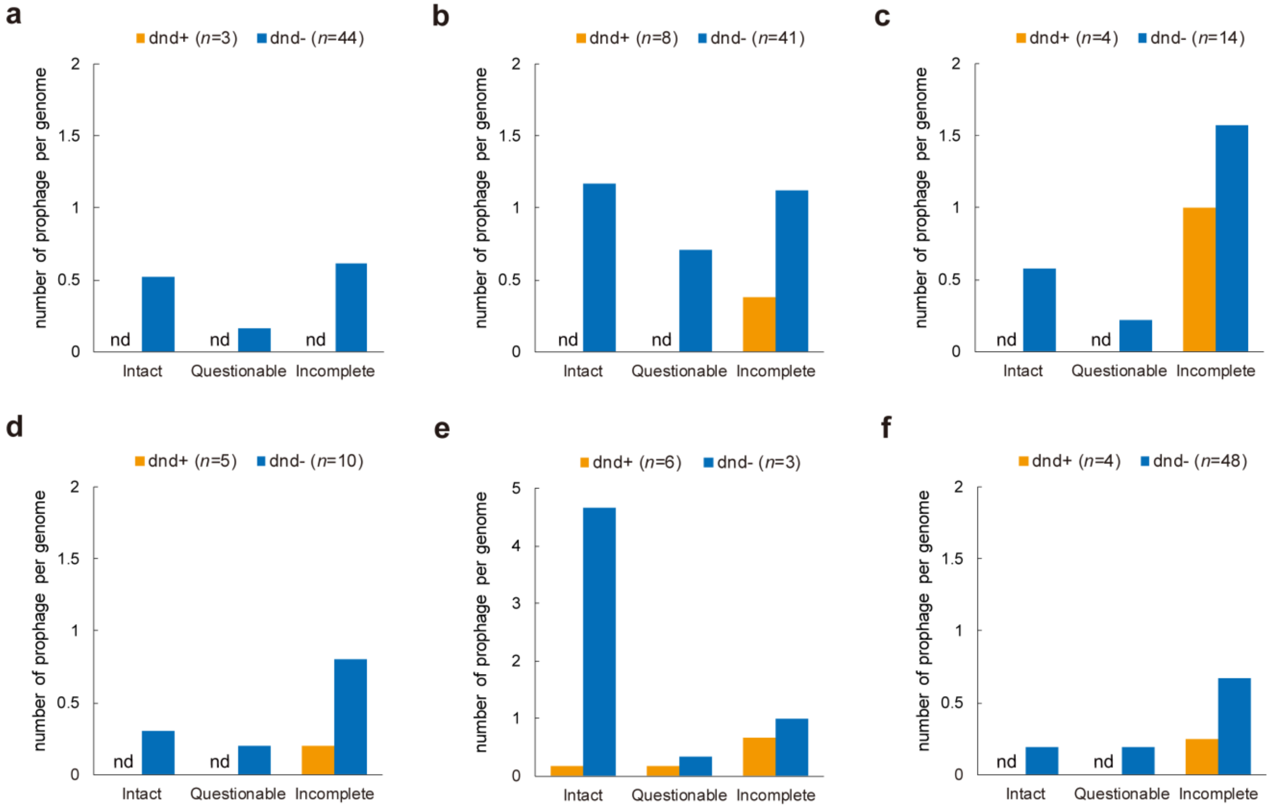

Fig. S7. Prophages in the *dnd*-positive strains are rare. Six different genera (**a**, *Shewanellaceae*; **b**, *Aeromonas*; **c**, *Halomonas*; **d**, *Marinobacter*; **e**, *Photobacterium*; **f**, *Rhodococcus*), which inhabited diverse environments, were chosen to analyse the relationship between the *dnd* gene cluster and prophages. The number of prophages in each bacterium was identified using the PHASTER tool[4]. The presence of a *dnd* gene cluster in each strain was determined according to a previous study[5].

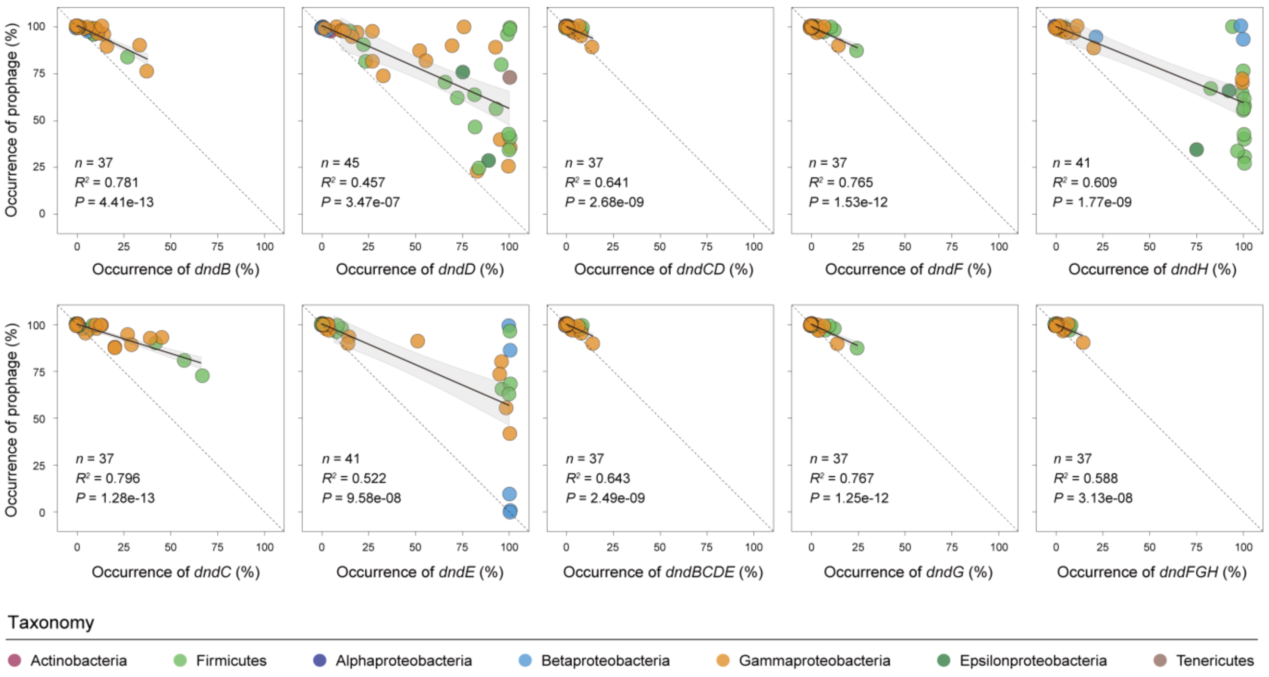

Fig. S8. Correlation analysis between the abundance of *dnd* genes/gene clusters and prophages among different species. The black solid line in each subplot refers to the best fitting, and the grey shadow displays the 95% confidence interval from linear regressions. The dashed lines depict the 1:1 linear relationship. The number of analysed species (*n*), *R* values and *P* values of linear regressions are shown in each subplot. Each circle represents a single species and is depicted in the color representing the taxonomy at the phylum and class levels (for Proteobacteria).

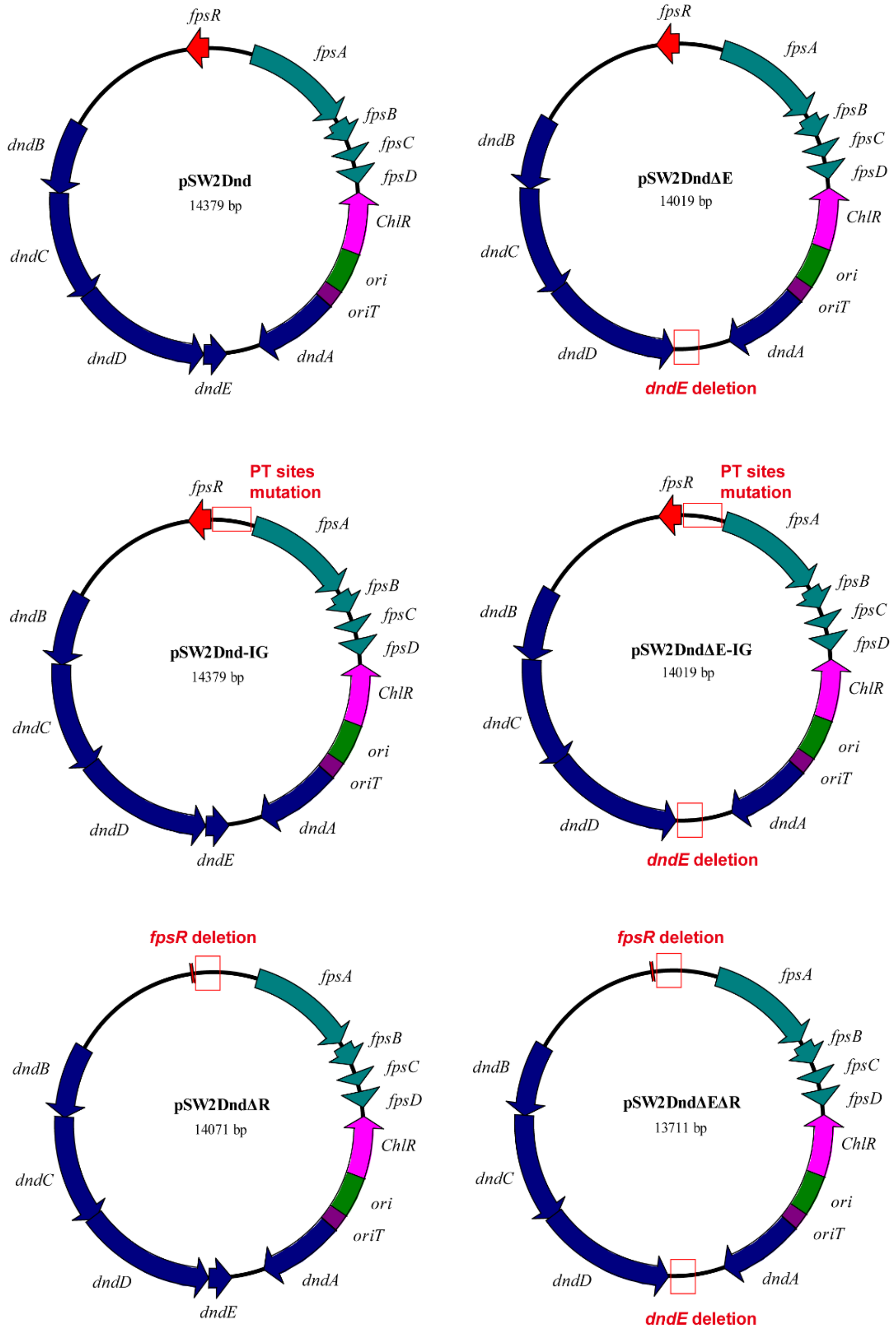

Fig. S9. Construction of the series of PT modification vectors based on pSW2Dnd[6]. A detailed method for the construction of vectors can be found in the Materials and Methods. The mutated components in each vector compared to pSW2Dnd are indicated by red boxes.

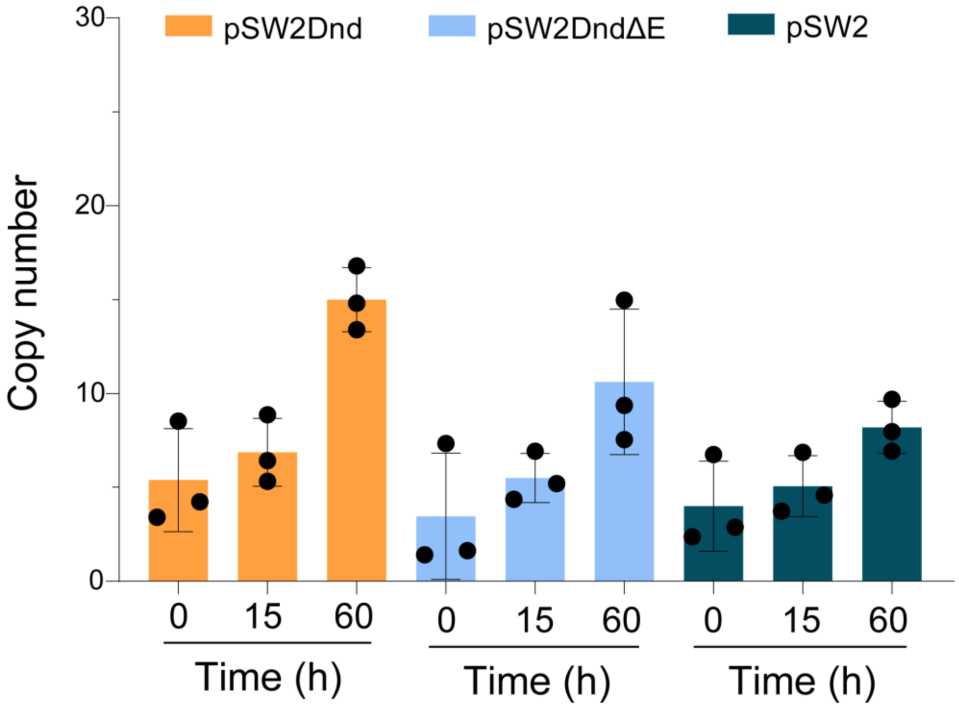

Fig. S10. Stability of different PT modification vectors. Samples were taken from cultures at different time points during the growth of WP3NR strains, and the DNA copy number was determined via qPCR. Data are represented as mean ± s.d.. and are based on three biologically independent samples.

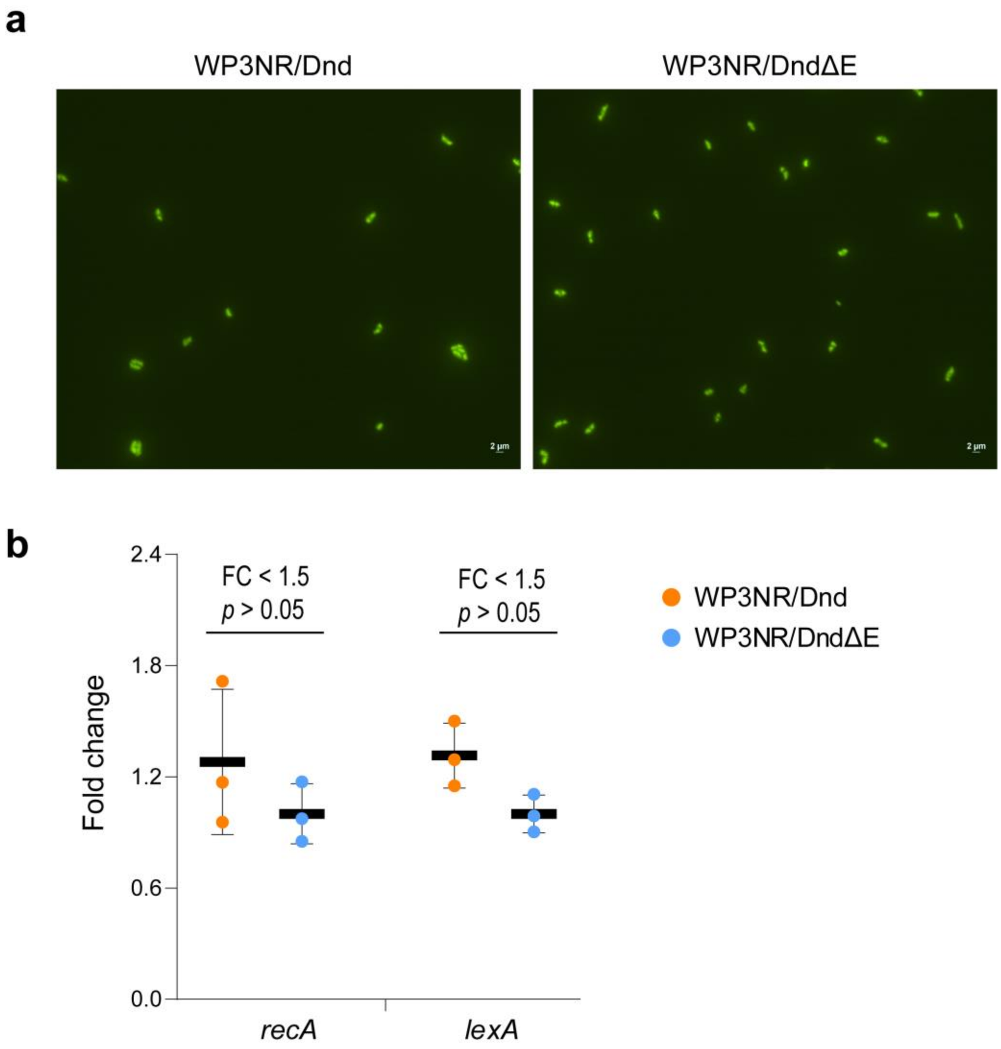

Fig. S11. Examination of the potential SOS response in WP3NR strains. **a.** Cell morphology of WP3NR/Dnd and WP3NR/DndΔE. The cells were stained with SYBR-Gold and visualized by epifluorescence microscopy. The scale bars are shown at the bottom right. Results are representative of two independent experiments. **b.** Relative transcriptional levels of the key SOS pathway genes *recA* and *lexA* in the PT-modified strain. The transcription level of the non-PT modified strain WP3NR/DndΔE was set as 1. Data are represented as mean ± s.d.. and are based on three biologically independent samples. The data were analysed by two-sided unpaired Student's *t* test. Specifically, P = 0.3162 for *recA* and P = 0.0546 for *lexA* in WP3NR/Dnd vs WP3NR/DndΔE.

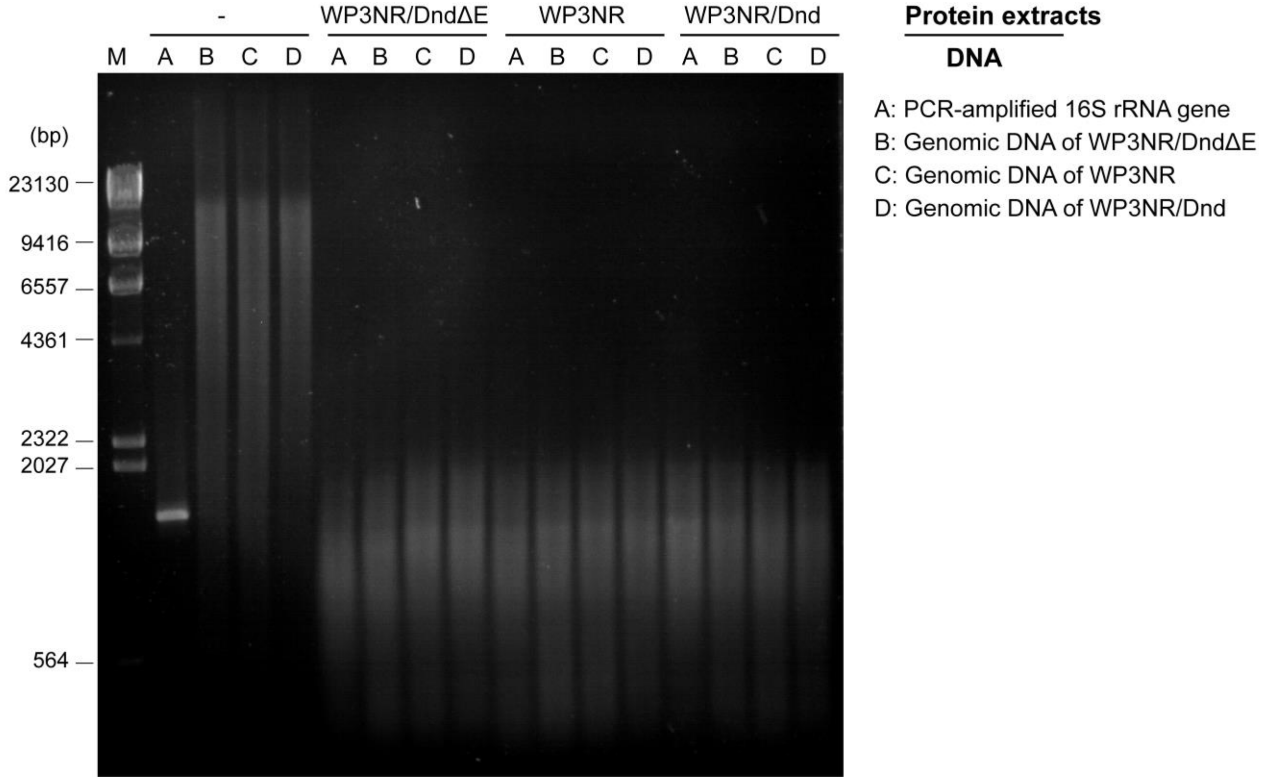

Fig. S12. Nuclease activity assay of WP3NR strains. Cell lysates derived from different bacterial cultures grown to the mid-exponential phase were assayed for their nuclease activity by the addition of DNA fragments of the 16S rRNA gene and genomic DNA of different strains. DNA without the addition of supernatants was used as a control. Images of representative agarose gels from two independent experiments are shown. M = Lambda DNA/HindIII marker (Fermentas).

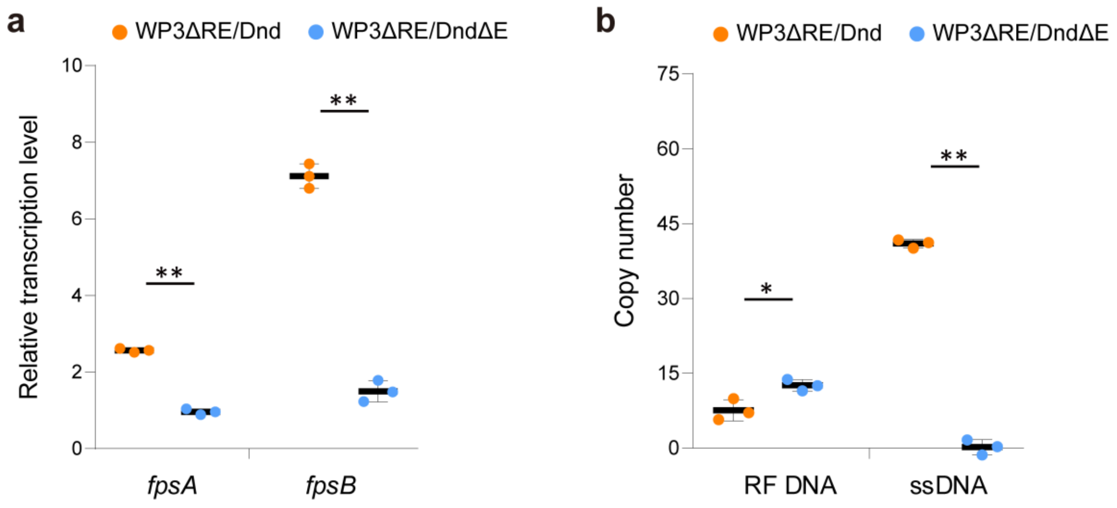

Fig. S13. PT modification influences the gene transcription and DNA replication of phage SW1. **a.** RTLs of SW1 genes in the WP3ΔRE/Dnd and WP3ΔRE/DndΔE strains. **b.** RF DNA and ssDNA copy numbers of SW1 in the WP3ΔRE/Dnd and WP3ΔRE/DndΔE strains. Data are represented as mean ± s.d.. and are based on three biologically independent samples. The data were analysed by two-sided unpaired Student's *t* test. Specifically, $P = 7.01\times10^{-6}$ (**) for *fpsA* and $P = 2.14\times10^{-5}$ (**) for *fpsB* in WP3ΔRE/Dnd vs WP3ΔRE/DndΔE; $P = 0.0233$ (*) for RF DNA and $P = 2.10\times10^{-6}$ (**) for ssDNA in WP3ΔRE/Dnd vs WP3ΔRE/DndΔE.

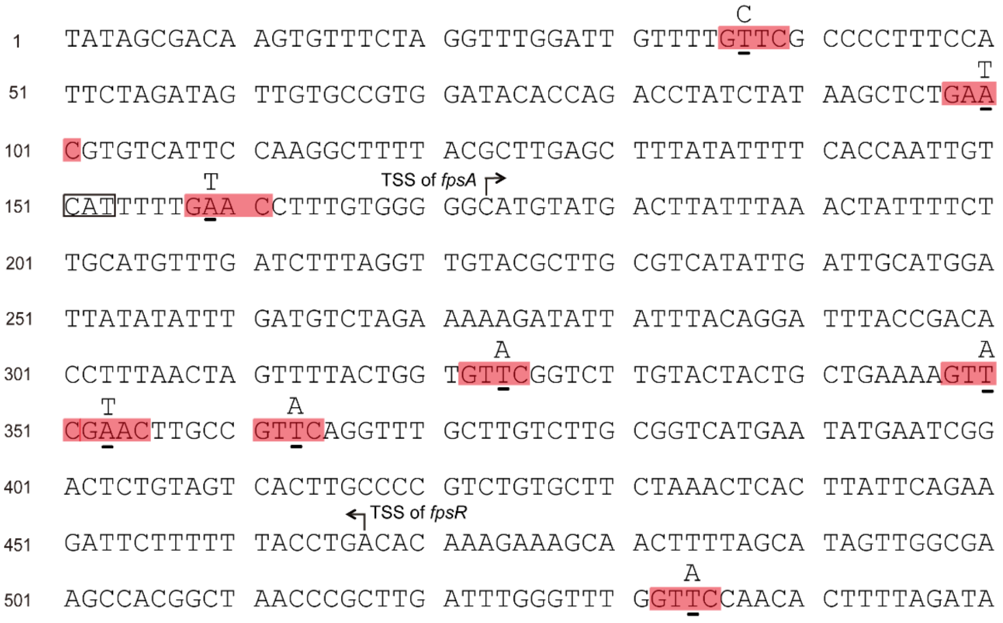

Fig. S14. Base substitution of PT motifs in the *fpsA*-*fpsR* intergenic region. The transcription start sites of the *fpsA* and *fpsR* genes are marked with angled arrows. The PT modification motifs are highlighted in red. The single-base mutations are underlined with solid lines, and the substituted bases are indicated above.

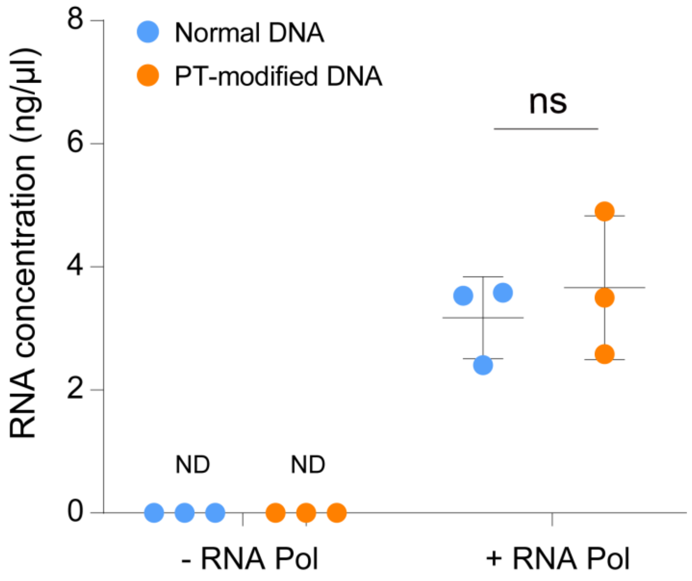

Fig. S15. In vitro transcription assay of PT-modified DNA and normal DNA. The influence of PT modification on RNA polymerase activity was assessed by quantifying the amount of synthesized RNA. Reaction mixtures lacking RNA polymerase addition were used as negative controls. ND, not detected. Data are represented as mean ± s.d.. and are based on three biologically independent samples. The data were analysed by two-sided unpaired Student's *t* test. Specifically, $P = 0.3631$ (ns, not significantly different) for the PT-modified and normal DNA.

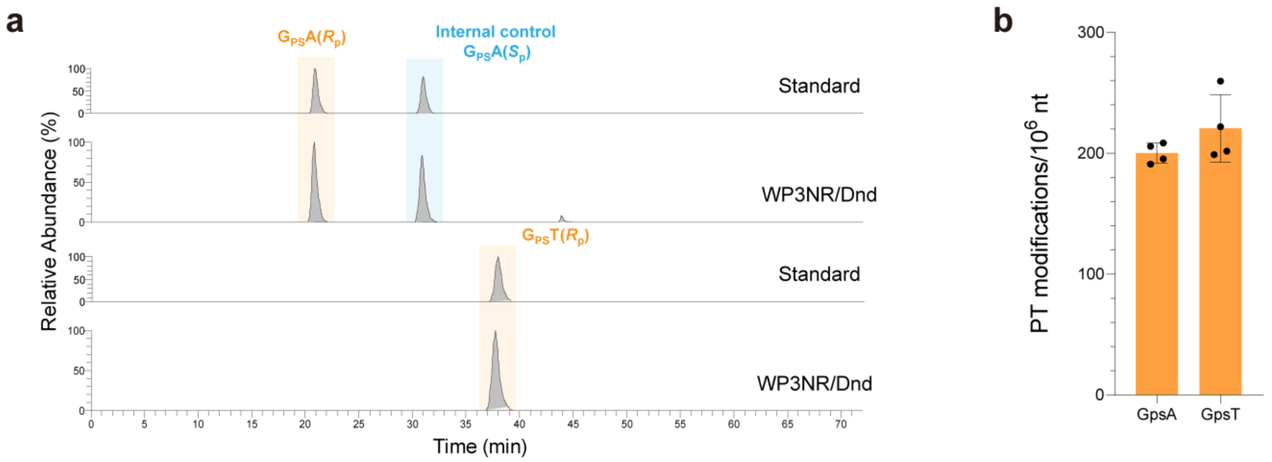

Fig. S16. **a**. PT modifications in WP3NR/Dnd quantified using LC-MS/MS. Chemically synthesized d($G_{ps}A$) and d($G_{ps}T$) in the R_p configuration were used as references. PT-linked dinucleotides were detected in the hydrolysate of WP3NR/Dnd genomic DNA. Representative results from four replicate assays are shown. **b**. Quantification of PT modifications ($G_{ps}A$ and $G_{ps}T$) in WP3NR/Dnd. Data are represented as mean ± s.d.. and are based on four biologically independent samples.

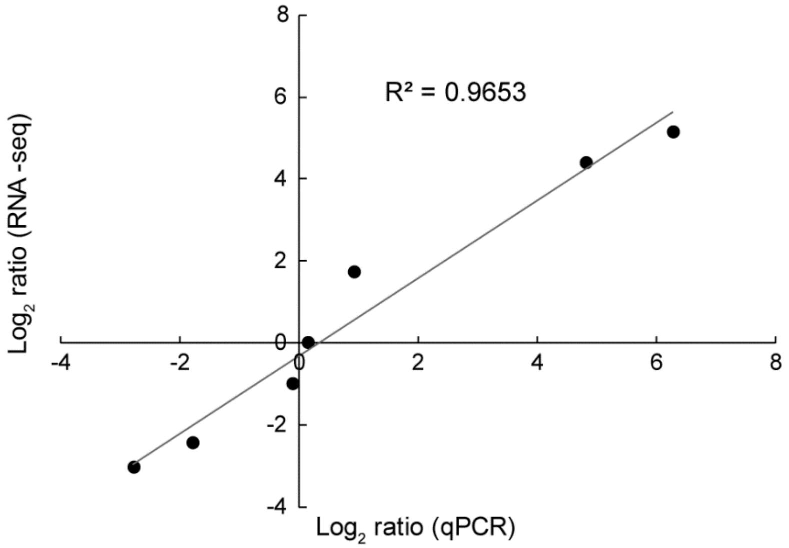

Fig. S17. Correlation analysis of the RNA-seq and RT-qPCR assays. Seven genes showing different expression levels were selected randomly for this assay. The RT-qPCR log_2 values were plotted against the RNA-seq log_2 values, and the correlation coefficient (R^2) is shown in the plot.

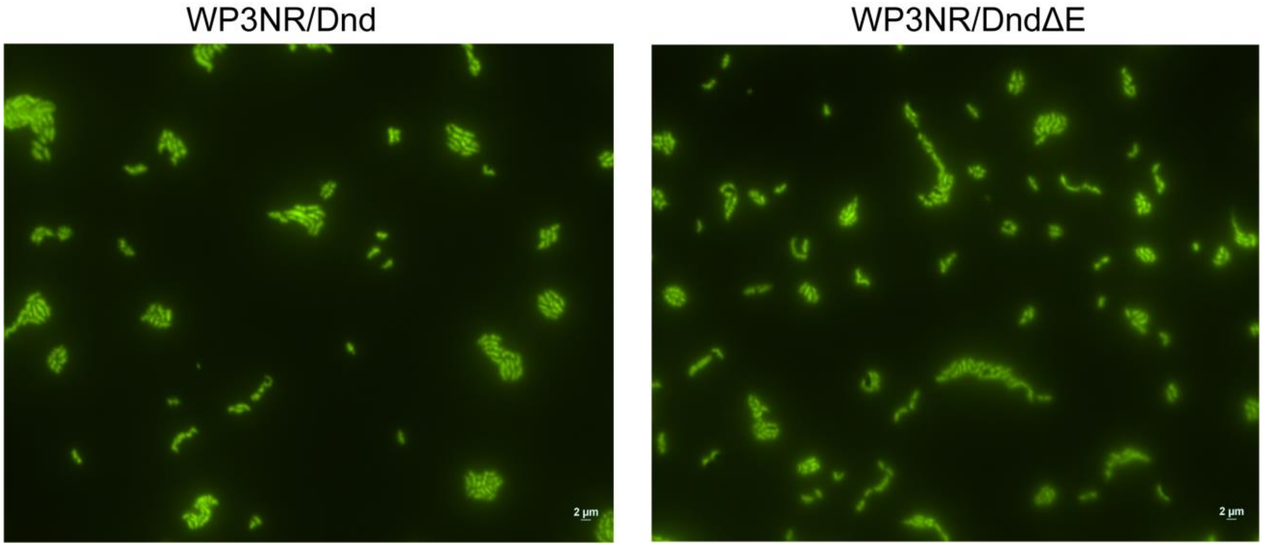

Fig. S18. Cell morphology of the PT-modified strain WP3NR/Dnd. The cells were stained with SYBR-Gold and visualized by epifluorescence microscopy. The scale bars are shown at the bottom right. Results are representative of two independent experiments.

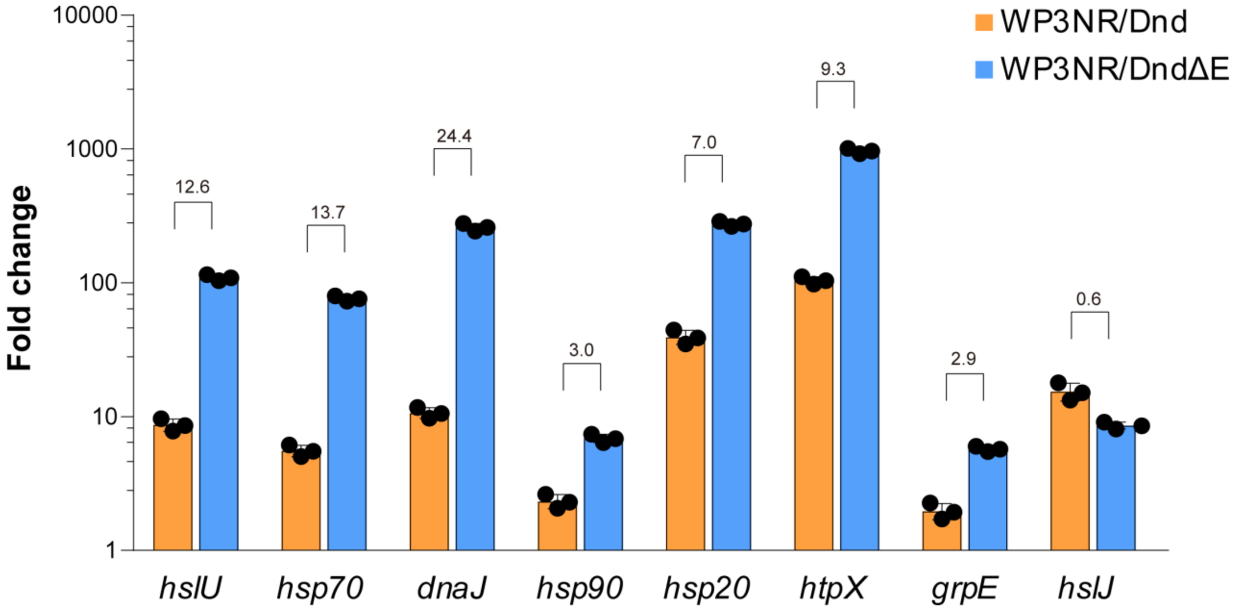

Fig. S19. PT modification influences the transcription of heat-shock genes in WP3 under high-temperature stress. The relative transcription levels of genes encoding heat shock proteins in the WP3NR/Dnd strain were compared with those in the WP3NR/DndΔE strain at 28°C. The transcription levels of these genes in the WP3NR/DndΔE strain at 20°C were set as 1. Data are represented as mean ± s.d.. and are based on three biologically independent samples.

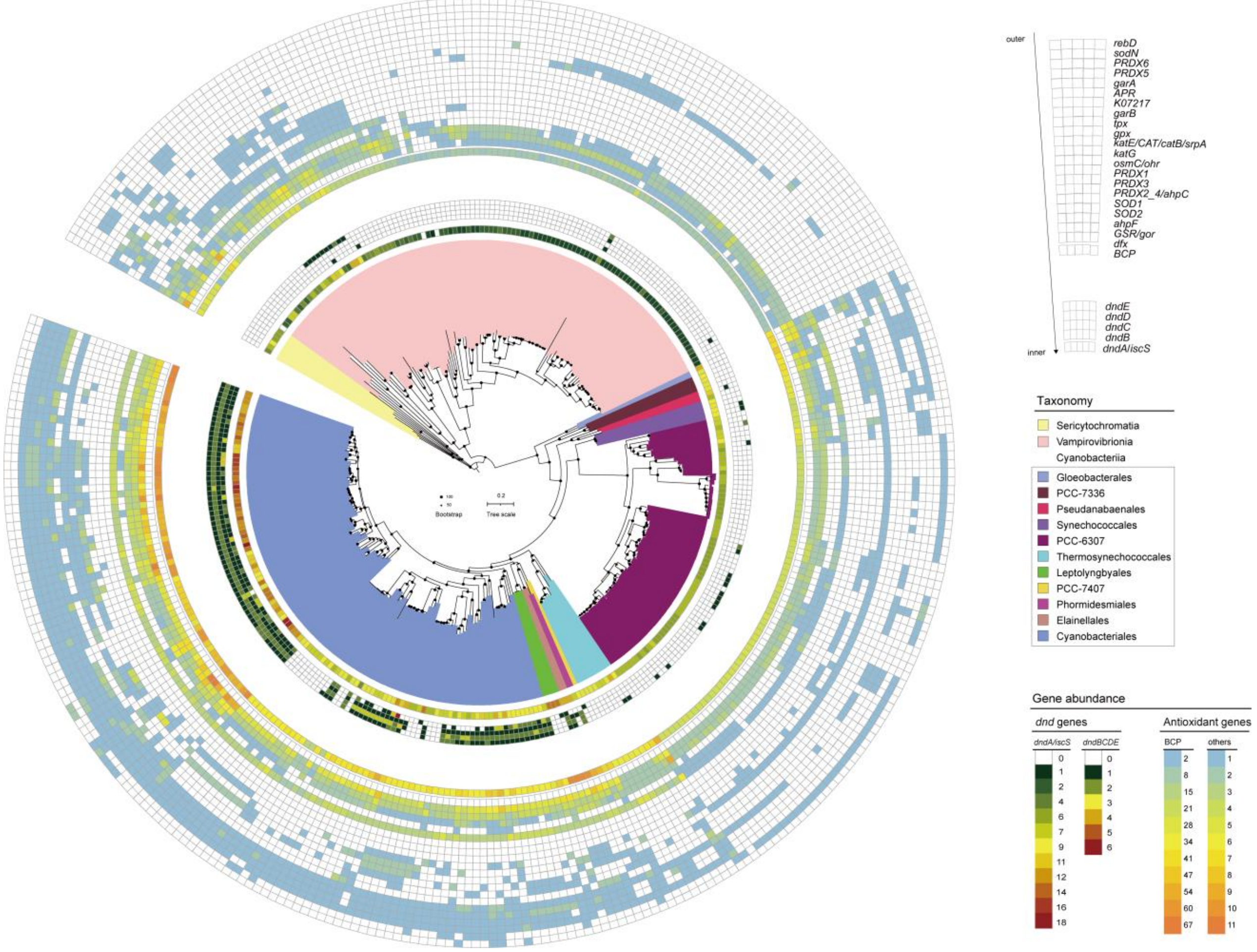

Fig. S20. Phylogenetic distribution of antioxidant genes in Cyanobacteria. The phylogenetic tree and distribution of *dnd* genes are shown in Fig. 2b in the main text. The antioxidant genes were identified based on their KEGG annotation. Antioxidant genes are abbreviated as follows: *BCP*, peroxiredoxin Q/BCP (K03564); *dfx*, superoxide reductase (K05919); *GSR/gor,* glutathione reductase (NADPH) (K00383); *ahpF*, alkyl hydroperoxide reductase subunit F (K03387); *SOD2*, superoxide dismutase (Fe-Mn family) (K04564); *SOD1*, superoxide dismutase (Cu-Zn family) (K04565); *PRDX2_4/ahpC*, peroxiredoxin (alkyl hydroperoxide reductase subunit C) (K03386); *PRDX3*, peroxiredoxin 3 (K20011); *PRDX1*, peroxiredoxin 1 (K13279); *osmC/ohr*, lipoyl-dependent peroxidase (K04063); *katG*, catalase-peroxidase (K03782); *katE/CAT/catB/srpA*, catalase (K03781); *gpx*, glutathione peroxidase (K00432); *tpx*, thiol peroxidase, atypical 2-Cys peroxiredoxin (K11065); *garB*, glutathione amide reductase (K23076); Mn-containing catalase (K07217); *APR*, adenylyl-sulfate reductase

(glutathione) (K05907); *garA*, glutathione amide-dependent peroxidase (K23075); *PRDX5*, peroxiredoxin 5, atypical 2-Cys peroxiredoxin (K11187); *PRDX6*, peroxiredoxin 6,1-Cys peroxiredoxin (K11188); *ahpD*, alkyl hydroperoxide reductase subunit D (K04756); *sodN*, nickel superoxide dismutase (K00518); *rebD*, dichlorochromopyrrolate synthase/catalase (K19885); *GPX4*, phospholipid-hydroperoxide glutathione peroxidase (K05361). The circular heatmap shows the abundance of *dnd* genes and antioxidant genes in Cyanobacteria genomes.

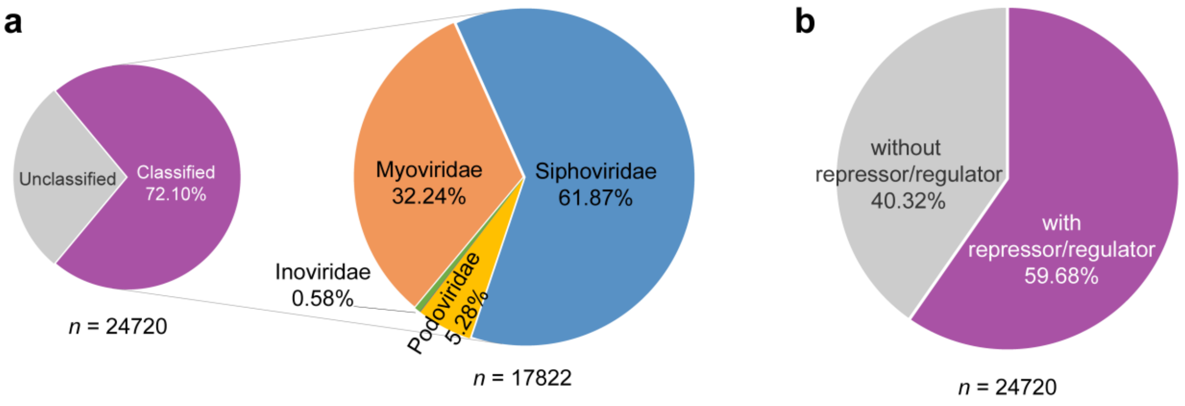

Fig. S21. a. Taxonomic classification of the prophages identified in bacterial genomes. The pie chart on the left indicates the percentage of prophages with taxonomic assignment (purple), and the pie graph on the right shows the relative abundance (%) of viral taxa at the family level for those prophages with predicted taxonomy. **b.** Occurrence of repressors and regulators encoded by prophages.

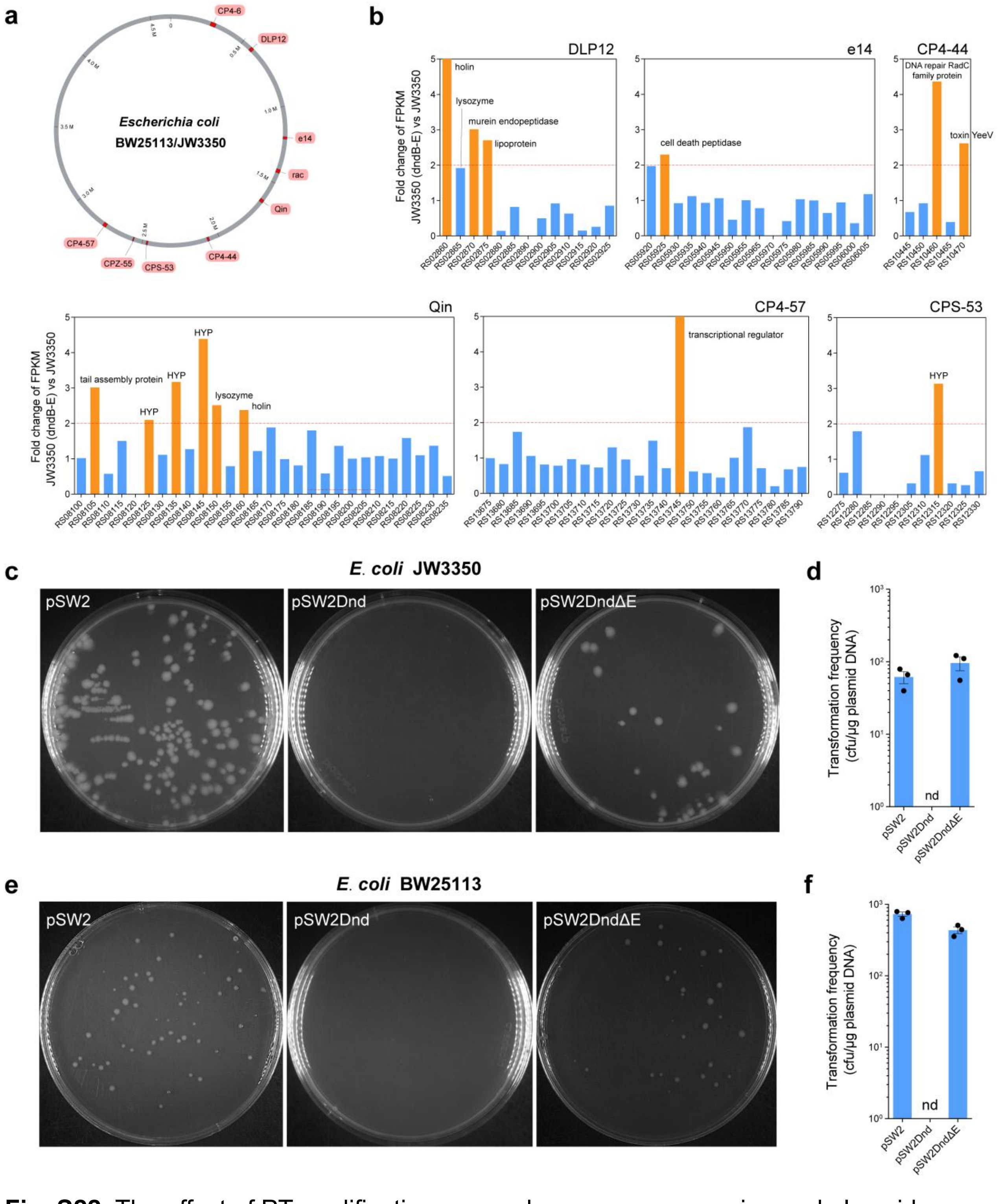

Fig. S22. The effect of PT modification on prophage gene expression and plasmid transfer of *E. coli*. **a**. The distribution of prophages (highlighted in red) in the *E. coli* BW25113 and JW3350 genomes. **b**. The expression levels of prophage genes in JW3350/Dnd compared with those in JW3350 measured by FPKM (fragments per kilobase per million). The red dashed line indicates a 2-fold change, which is considered

the threshold for differentially expressed genes. The publicly available transcriptomic data were obtained from a previous study (accession number GSE135938 in the GEO database)[7]. **c-f**. Transformation of pSW2Dnd and pSW2DndΔE to *E. coli* JW3350 (**c**-**d**) and BW25113 (**e**-**f**). The transformation frequency was calculated as the number of transformants per μg plasmid DNA. nd: not detected in the current assays (transformation frequency < 1.1×10^1). Data are represented as mean ± SEM and are based on three independent experiments.

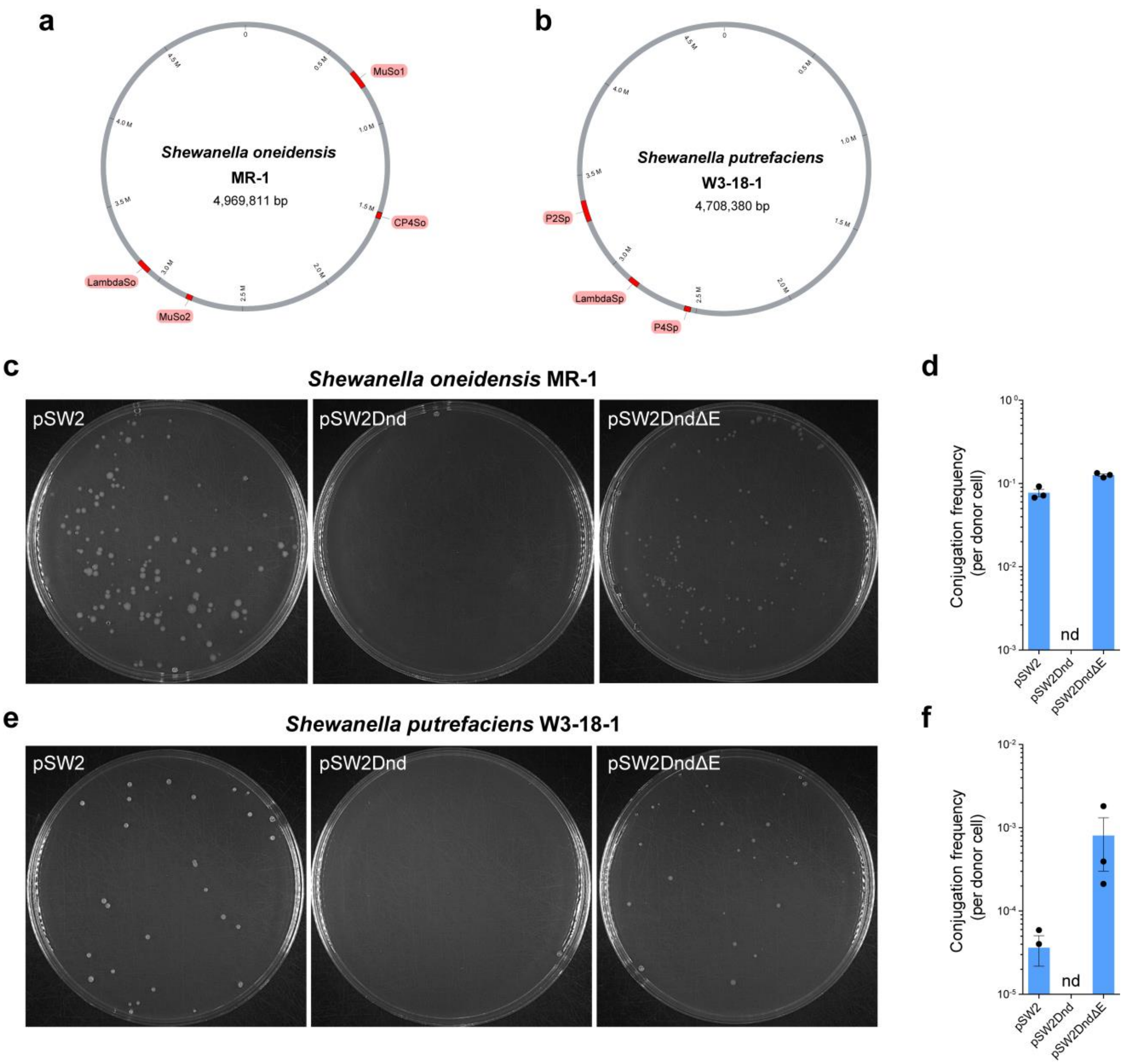

Fig. S23. a-b. Conjugation assay of plasmids pSW2Dnd and pSW2DndΔE to *Shewanella* strains. **a**-**b**. The distribution of prophages (highlighted in red) in the *S. oneidensis* MR-1 (**a**) and *S. putrefaciens* W3-18-1 (**b**) genomes. **c**-**f**. Conjugal transfer of pSW2Dnd and pSW2DndΔE to *S. oneidensis* MR-1 (**c**-**d**) and *S. putrefaciens* W3-18-1 (**e**-**f**). The *E. coli* strain WM3064 harbouring pSW2Dnd or pSW2DndΔE was used as the donor. As a control, the WM3064 strain harbouring pSW2 was used. The conjugation frequency was calculated as the number of transconjugants per number of donors. nd: not detected in the current assays (conjugation frequency < 1.1×10^{-7}). Data are represented as mean ± SEM and are based on three independent experiments.

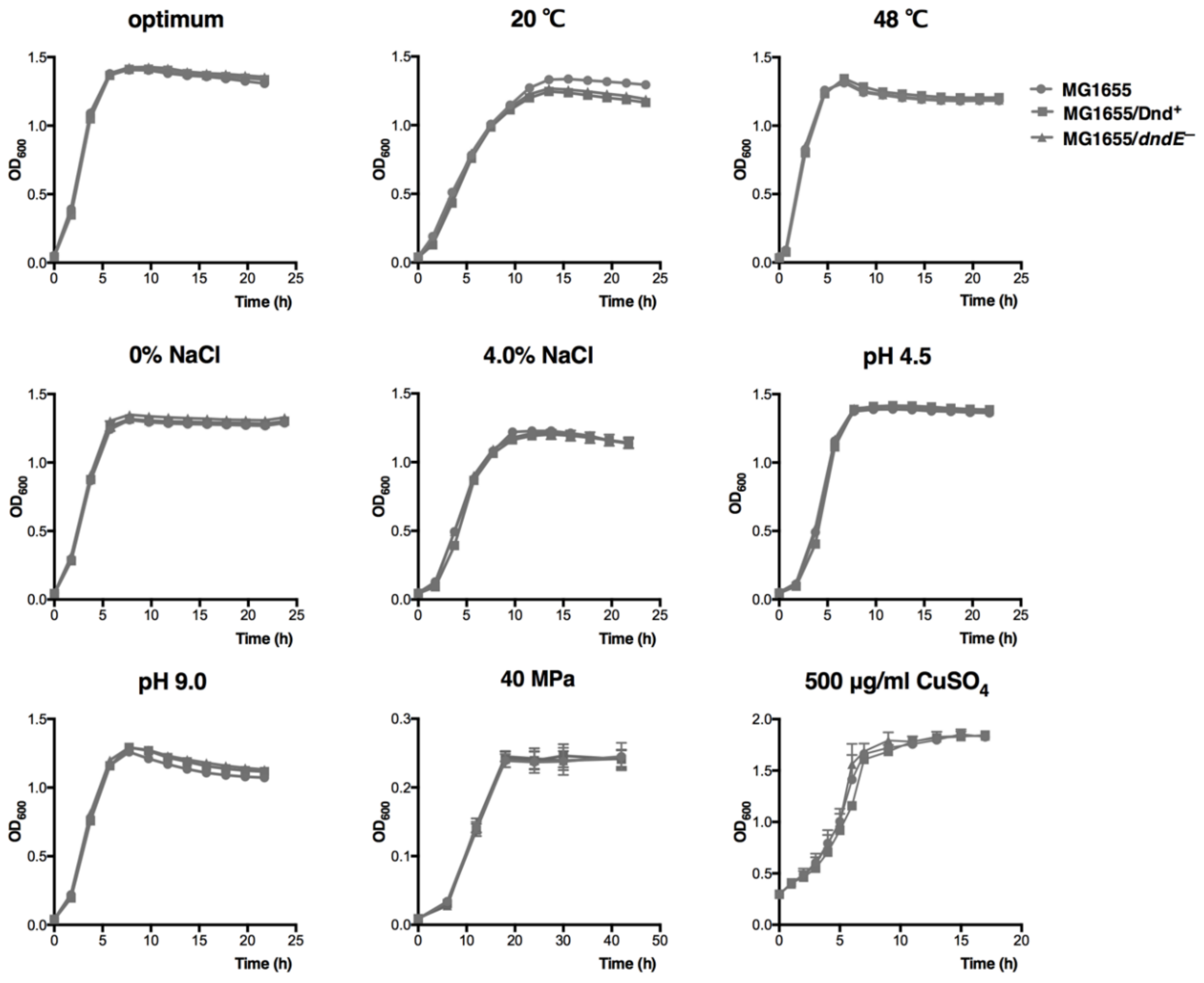

Fig. S24. Growth curves of *E. coli* MG1655 PT strains under multiple stress conditions. The growth of the strains was detected at OD_{600nm}. Data are represented as mean ± s.d. and are based on three biologically independent samples.

Table S1. Correlation analysis of the occurrence of *dnd* gene/gene clusters and prophages at the genus/species level. Pearson correlation coefficient was calculated via the Python function “Pearsonr” from SciPy (v1.0) and linear regression analyses in R (v3.5.3). Two-sided unpaired Student *t* test was used to measure significance (*p* value). No adjustments were made for multiple comparisons.

	**Gene/gene cluster**	**Pearson correlation coefficient**	***p* value**	**Number of genus/species**	**Number of genomes**
**genus**	*dndB*	-0.916	4.80E-13	*n*=31	13565
	dndC	-0.753	1.04E-06	*n*=31	13565
	dndD	-0.737	1.53E-06	*n*=32	13693
	dndE	-0.792	1.09E-07	*n*=31	13565
	dndCD	-0.800	6.45E-08	*n*=31	13565
	dndBCDE	-0.877	9.56E-11	*n*=31	13565
	dndF	-0.876	1.13E-10	*n*=31	13565
	dndG	-0.784	1.81E-07	*n*=31	13565
	dndH	-0.783	1.89E-07	*n*=31	13565
	dndFGH	-0.789	1.31E-07	*n*=31	13565
**species**	*dndB*	-0.884	4.41E-13	*n*=37	8334
	dndC	-0.892	1.28E-13	*n*=37	8334
	dndD	-0.676	3.47E-07	*n*=45	8749
	dndE	-0.723	9.58E-08	*n*=41	8547
	dndCD	-0.801	2.68E-09	*n*=37	8334
	dndBCDE	-0.802	2.49E-09	*n*=37	8334
	dndF	-0.875	1.53E-12	*n*=37	8334
	dndG	-0.876	1.25E-12	*n*=37	8334
	dndH	-0.781	1.77E-09	*n*=41	8499
	dndFGH	-0.767	3.13E-08	*n*=37	8334

Table S2. Kinetic parameters for the interaction between FpsR and PT-modified/normal DNA[a].

	**Normal DNA**	**PT-modified DNA**
K_a (1/ms)	6.32E+05	8.64E+05
K_a T value	9.27E+01	9.46E+01
K_a SE	6.82E+03	9.32E+03
K_d (1/s)	4.43E-03	3.28E-03
K_d T value	2.35E+02	2.31E+02
K_d SE	1.89E-05	1.42E-05
K_D (M)	7.01E-09	3.80E-09
K_D SE	6.61E-21	1.95E-21

[a] Equilibrium dissociation constants (K_D) were determined by surface plasmon resonance (SPR) using a Biacore 8K system. Errors listed in this table are the standard errors for the fit to a Langmuir 1:1 binding model. K_a, association rate constant; K_d, dissociation rate constant.

Table S3. Relative fitness of PT-modified *S. piezotolerans* WP3 strain as measured by competition assays.

Temperature	Sample	Number of colonies	WP3NR/Dnd (%) [a]	WP3NR/DndΔE (%) [a]	Relative fitness
28°C	T0	212±13	50.67	49.33	1.1797±0.0468
	T1	202±14	33.67	66.33	1.1308±0.0128
	T5	171±23	6.67	93.33	1.1189±0.0051
20°C	T0	329±26	52.33	47.67	1.0382±0.0272
	T1	345±27	48	52	1.0421±0.0031
	T5	352±31	31	69	1.0431±0.0102

[a]The average percentage of each strain identified by PCR (100 colonies per plate).

Table S4. Differentially expressed genes in WP3NR/Dnd by compared with WP3NR/DndΔE. Data represents the averaged fold change (FC) of transcription level and are based on three biologically independent samples. The *P*-value corresponding to differential gene expression test was calculated by edgeR based on an overdispersed Poisson model[8]. False Discovery Rate (FDR) was used in the multiple hypothesis testing to correct for *P*-value by the Benjamini-Hochberg (BH) method.

Gene ID	log2FC	*P*-value	FDR	Protein ID	Description
SWP_RS13835	-3.0364	0.00E+00	0.00E+00	WP_079891946.1	marine proteobacterial sortase target protein
SWP_RS13840	-3.0283	0.00E+00	0.00E+00	WP_020913159.1	OmpA
SWP_RS13830	-2.8235	2.04E-251	2.08E-248	WP_052634186.1	class GN sortase
SWP_RS11570	-2.4380	6.40E-10	2.31E-08	WP_020912663.1	zonular occludens toxin
SWP_RS14445	-2.0831	6.25E-03	4.39E-02	WP_020913285.1	hypothetical protein
SWP_RS19905	-1.9564	2.39E-191	1.62E-188	WP_020914447.1	class D beta-lactamase
SWP_RS22130	-1.7412	4.44E-18	2.92E-16	WP_020914944.1	hypothetical protein
SWP_RS20105	-1.5664	8.26E-158	4.81E-155	WP_020914486.1	porin
SWP_RS20440	-1.5651	1.52E-156	7.76E-154	WP_020914562.1	TonB-dependent receptor
SWP_RS13630	-1.3941	1.61E-03	1.44E-02	WP_020913110.1	hypothetical protein
SWP_RS19760	-1.3370	4.25E-21	3.27E-19	WP_020914413.1	purine-nucleoside phosphorylase
SWP_RS10565	-1.3175	5.30E-194	4.32E-191	WP_020912467.1	TonB-dependent receptor
SWP_RS17750	-1.2992	2.00E-12	9.07E-11	WP_020913987.1	heme utilization cystosolic carrier protein HutX
SWP_RS13850	-1.2741	3.34E-39	5.05E-37	WP_044555935.1	proteobacterial dedicated sortase system histidine kinase
SWP_RS13845	-1.2691	1.05E-59	2.86E-57	WP_020913160.1	proteobacterial dedicated sortase system response regulator
SWP_RS05840	-1.2688	2.52E-04	3.08E-03	WP_020911487.1	hypothetical protein
SWP_RS09445	-1.2359	1.01E-08	3.05E-07	WP_020912232.1	NAD(P)/FAD-dependent oxidoreductase
SWP_RS17240	-1.1983	2.84E-46	5.52E-44	WP_020913886.1	hypothetical protein

SWP_RS04225	-1.1928	1.53E-55	3.47E-53	WP_020911140.1	CBS domain-containing protein
SWP_RS08090	-1.1521	7.14E-04	7.25E-03	WP_044555782.1	tol-pal system-associated acyl-CoA thioesterase
SWP_RS08380	-1.1447	5.44E-04	5.85E-03	WP_020912028.1	azurin
SWP_RS21235	-1.1204	3.66E-09	1.19E-07	WP_020914743.1	hypothetical protein
SWP_RS22125	-1.0629	6.20E-25	5.62E-23	WP_020914943.1	hypothetical protein
SWP_RS22120	-1.0619	6.08E-57	1.46E-54	WP_020914942.1	DNA-binding response regulator
SWP_RS02595	-1.0458	4.61E-03	3.47E-02	WP_052634156.1	hypothetical protein
SWP_RS22005	-1.0385	5.59E-03	4.03E-02	WP_020914918.1	VUT family protein
SWP_RS11335	-1.0324	1.00E-30	1.24E-28	WP_020912616.1	bifunctional metallophosphatase/5'-nucleotidase
SWP_RS02455	-1.0036	5.56E-04	5.96E-03	WP_020910746.1	hypothetical protein
SWP_RS17785	-1.0010	5.24E-03	3.85E-02	WP_044556508.1	heme ABC transporter ATP-binding protein
SWP_RS20550	1.0133	6.73E-03	4.64E-02	WP_020914585.1	STAS/SEC14 domain-containing protein
SWP_RS18475	1.0359	7.94E-08	2.04E-06	WP_020914144.1	Probable cytochrome-c peroxidase
SWP_RS17675	1.0647	1.98E-60	5.76E-58	WP_020913969.1	ammonia-forming nitrite reductase cytochrome c552 subunit
SWP_RS13815	1.0943	1.68E-04	2.15E-03	WP_020913154.1	hypothetical protein
SWP_RS15855	1.0978	3.39E-67	1.15E-64	WP_020913587.1	TonB-dependent receptor
SWP_RS02860	1.1086	6.40E-03	4.46E-02	WP_020910846.1	chitinase
SWP_RS07595	1.1212	5.15E-67	1.62E-64	WP_020911855.1	TonB-dependent receptor
SWP_RS16275	1.1471	1.25E-06	2.56E-05	WP_020913687.1	OsmC family peroxiredoxin
SWP_RS11885	1.1614	6.50E-37	9.14E-35	WP_044555880.1	hydrogenase
SWP_RS17950	1.2004	1.36E-75	5.05E-73	WP_020914033.1	TonB-dependent receptor
SWP_RS01970	1.2848	2.03E-06	4.00E-05	WP_020910642.1	fumarate reductase respiratory complex%2C subunit
SWP_RS19980	1.3404	1.22E-131	5.55E-129	WP_020914464.1	lipase family protein
SWP_RS19985	1.3572	1.18E-78	4.82E-76	WP_020914465.1	hypothetical protein
SWP_RS19990	1.3596	1.00E-11	4.27E-10	WP_020914466.1	hypothetical protein
SWP_RS11310	1.3992	1.19E-06	2.45E-05	WP_020912611.1	FAD:protein FMN transferase

SWP_RS19975	1.4061	1.04E-54	2.24E-52	WP_020914463.1	DUF1508 domain-containing protein
SWP_RS02420	1.4293	2.46E-07	5.98E-06	WP_044555588.1	hypothetical protein
SWP_RS05470	1.5297	2.10E-04	2.62E-03	WP_020911408.1	DNA-binding response regulator
SWP_RS09400	1.5584	3.51E-03	2.77E-02	WP_044555807.1	hypothetical protein
SWP_RS17205	1.5867	6.38E-03	4.45E-02	WP_020913879.1	RNA polymerase sigma factor
SWP_RS07215	1.6129	1.16E-03	1.10E-02	WP_048908459.1	hypothetical protein
SWP_RS09565	1.7216	1.15E-04	1.53E-03	WP_044555815.1	LysR family transcriptional regulator
SWP_RS11920	1.7720	2.47E-03	2.08E-02	WP_020912737.1	HypC/HybG/HupF family hydrogenase formation chaperone
SWP_RS15615	1.8168	4.01E-04	4.51E-03	WP_020913538.1	NADPH:quinone reductase and related Zn-dependent oxidoreductase
SWP_RS04865	2.0237	1.69E-53	3.44E-51	WP_020911283.1	hypothetical protein
SWP_RS04770	2.6177	1.64E-03	1.47E-02	WP_020911263.1	hypothetical protein
SWP_RS17130	4.3937	1.02E-45	1.90E-43	WP_044556498.1	alkaline phosphatase
SWP_RS17125	5.1544	0.00E+00	0.00E+00	WP_044556048.1	TonB-dependent receptor

Table S5. Bacterial strains and plasmids used in this study.

Strain or plasmid	Relevant genotype	Reference or source
E. coli strains		
WM3064	Donor strain for conjugation; Δ*dapA*	Lab stock
C41(DE3)	Recombinant protein expression host	GE healthcare
BW25113	*E. coli* K-12 derivative	[9]
JW3350	*E. coli* BW25113 Δ*dam* mutant	[9]
MG1655	*E. coli* K-12 wild type strain	[10]
MG1655/Dnd+	MG1655 strain harboring pJTU1238	This work
MG1655/Dnd-	MG1655 strain harboring pJTU3529	This work
Shewanella strains		
MR-1	*S. oneidensis* MR-1 wild type strain	Dr. Xiaoxue Wang
W3-18-1	*S. putrefaciens* W3-18-1 wild type strain	Dr. Xiaoxue Wang
S. piezotolerans WP3 strains		
WP3	*S. piezotolerans* WP3 wild type strain	[11]
WP3NR	Wild-type WP3 strain with 3 endonuclease-encoding genes (*swp0009*, *swp0840*, *swp2190*) and phage SW1 deletion	[6]
WP3NR/Dnd	WP3NR strain harboring pSW2Dnd	[6]
WP3NR/DndΔE	WP3NR strain harboring pSW2DndΔE	This work
WP3NR/Dnd-IG	WP3NR strain harboring pSW2Dnd-IG	This work
WP3NR/DndΔE-IG	WP3NR strain harboring pSW2DndΔE-IG	This work
WP3NR/DndΔR	WP3NR strain harboring pSW2DndΔR	This work
WP3NR/DndΔEΔR	WP3NR strain harboring pSW2DndΔEΔR	This work
WP3ΔRE	Wild-type WP3 strain with 3 endonuclease-encoding genes (*swp0009*, *swp0840*, *swp2190*) deletion	Lab stock
WP3ΔRE/Dnd	WP3ΔRE strain harboring pSW2Dnd	This work
WP3ΔRE/DndΔE	WP3ΔRE strain harboring pSW2DndΔE	This work
Plasmids		
pSW2	Chl^r, derived from the filamentous bacteriophage SW1	[12]
pSW3	pSW2 containing *pepN*, used for SW1 RF- and ssDNA quantification	[13]
pET24b	Kan^r, His-tag protein expression vector	Novagen
pJTU1238	pBluescript II sk (+) derivative carrying a 6.7 kb fragment of *dndBCDE* gene cluster from *S. enterica* Cerro 87, Amp^r	[14]
pJTU3529	A derivative of pJTU1238, *dndE*$^-$, in-frame deletion, Amp^r	[15]
pJTU3619	pET15b carrying *dndA* from *E. coli*	[16]
pSW2Dnd	pSW2 derivative carrying *dndA* from pJTU3619 and *dndBCDE* from pJTU1238	[6]
pSW2DndΔE	pSW2Dnd with deletion of *dndE* gene	This work
pSW2Dnd-IG	pSW2Dnd with mutation of PT sites in the intergenic region between *fpsA* and *fpsR*	This work
pSW2DndΔE-IG	pSW2DndΔE with mutation of PT sites in the intergenic region between *fpsA* and *fpsR*	This work
pSW2DndΔR	pSW2Dnd with deletion of *fpsR* gene	This work

pSW2DndΔEΔR	pSW2DndΔE with deletion of *fpsR* gene	This work
pET24b-*fpsR*	pET24b containing the coding region of the *fpsR* gene	This work

Table S6. Primers and oligonucleotides used in this study.

Name	Sequence (5'-3')	Description
pSW2dndFor	CTCGAGATGAAATTACCGATTTATCTCGACTAC	Vector construction
pSW2dndRev	CTCGAGGCCAGCCTCGCAGAGCAGGATTCC	Vector construction
dndBczFor	ATGGCTAGTGTTGATGCAGA	Vector construction
dndBczRev	TCTGCATCAACACTAGCCAT	Vector construction
KOdndEFor	CTGTCTGATGTAGAATAATATGGCACCATGAA	Vector construction
KOdndERev	TATTATTCTACATCAGACAGCCTCCTTGGTT	Vector construction
SW1IGczFor	CTAGATTTTCTGAGCTGCTGATCAT	Vector construction
SW1IGczRev	CGAGCGGACGCAGACTCAACCGA	Vector construction
SW1IGdoczFor	GTTGAGTCTGCGTCCGCTCGACTTA	Vector construction
SW1IGupczRev	GCAGCTCAGAAAATCTAGAAAACGG	Vector construction
pSW2dndczFor	CAGGGAACCGGCCGCACTGCA	Vector construction
pSW2dndczRev	GTAATTTCATCTCGAGGCCAGCCTC	Vector construction
KOdndEyzFor	ATCAGGTTGTGCTGCTATCCA	Vector construction
KOdndEyzRev	ACTAATGTTAGGAGTTTGATTTACC	Vector construction
KOfpsRyzFor	TACATTGTGCGAATTCCTCTCGA	Vector construction
KOfpsRyzRev	CAGTAGTACAAGACCGAACACCA	Vector construction
pSW2seqFor	AGCGGGTGTTCCTTCTTCACTG	Vector construction
pSW2seqFev	CCTAATCTTCTGCGACCTCTGG	Vector construction
pSW2For	GCCCCTTAAGCGCTTATTATCACTTATTCA	Plasmid verification
pSW2Rev	TATGGAAAATAAATAAATCCTGGTGTCC	Plasmid verification
SW1RFRTFor	CACGCCATACGTTAATGAGTCTCT	qPCR
SW1RFRTRev	GACGGCCAGTATTCAACATAACAT	qPCR

pepNRTFor	TTAAGGCAATGGAAGCTGCAT	qPCR
pepNRTRev	CGTCTTTACCCGTTAATGATACGA	qPCR
tonRTFor	TGCCAGTAGTGGTGGCTCTCTT	qPCR
tonRTRev	TGAGCTTGCACTGTCGGTAAA	qPCR
alkRTFor	GGCCACAGCAACGATATTAGC	qPCR
alkRTRev	GACCGTCTTCGCCAAATCAT	qPCR
lysRTFor	AACCGCGAAATTGGCAACT	qPCR
lysRTRev	AATGCGGCCGAATGATTG	qPCR
hemRTFor	CGCCGACAGGCTAATCGTT	qPCR
hemRTRev	CGGCGATCCATCACTGACT	qPCR
zonRTFor	GTCAAGGCGTTTTATTTGTTGTTG	qPCR
zonRTRev	CTTGGCGTCACGGCCTAT	qPCR
marRTFor	AAGCAAACGGTGGCACTGA	qPCR
marRTRev	TCCTGCTGAGACAGCGCTTT	qPCR
gluRTFor	TCCACCTAAACTTGCGGCTTA	qPCR
gluRTRev	TCGCCATCGCCTTACGA	qPCR
fpsARTFor	GGCCACAACGCTACAATGG	qPCR
fpsARTRev	TGTTGCGCCTTTTCCTTAGCT	qPCR
fpsBRTFor	CCGACCCAAACGGTAGATGT	qPCR
fpsBRTRev	CCTGTAGCGTTTTAGGGTCTTTCA	qPCR
recARTFor	GCAGCAGCGCAGAAGCA	qPCR
recARTRev	GATCCAAGGCATGTTCTGCAT	qPCR
lexARTFor	GGAACCGGAAGAGGTCGAA	qPCR
lexARTRev	GCTCACCTGCAGCAACTTGA	qPCR
O4(PT)-F	5'-Biotin-TTTTACGCTTGAGCTTTATATTTTCACCAATTGTCATTTTTG*AACCTTTGTGGGGGC	SPR

O4(PT)-R	GCCCCCACAAAGG*TTCAAAAATGACAATTGGTGAAAATATAAAGCTCAAGCGTAAAA	SPR
O4-F	5’-Biotin-TTTTACGCTTGAGCTTTATATTTTCACCAATTGTCATTTTTGAACCTTTGTGGGGGC	SPR
O4-R	GCCCCCACAAAGGTTCAAAAATGACAATTGGTGAAAATATAAAGCTCAAGCGTAAAA	SPR
O4-fpsA(PT)-F	GGCTTTTACGCTTGAGCTTTATATTTTCACCAATTGTCATTTTTG*AACCTTTGTGGGGGCATGT ATGACTTATTTAAACTATTTTCTTGCATGTTTGATCTTTAGGTTGTACGCTTGCGTCATATTGATT	In vitro transcription
O4-fpsA(PT)-R	AATCAATATGACGCAAGCGTACAACCTAAAGATCAAACATGCAAGAAAATAGTTTAAATAAGTCAT ACATGCCCCCACAAAGG*TTCAAAAATGACAATTGGTGAAAATATAAAGCTCAAGCGTAAAAGCC	In vitro transcription
O4-fpsA-F	GGCTTTTACGCTTGAGCTTT	In vitro transcription
O4-fpsA-R	AATCAATATGACGCAAGCG	In vitro transcription

*, PT modification site

References

1. Chaumeil P-A, Mussig AJ, Hugenholtz P, Parks DH. GTDB-Tk: a toolkit to classify genomes with the Genome Taxonomy Database. *Bioinformatics* **36**, 1925-1927 (2020).

2. Price MN, Dehal PS, Arkin AP. FastTree 2 – Approximately Maximum-Likelihood Treesfor Large Alignments. *PLoS One* **5**, e9490 (2010).

3. Letunic I, Bork P. Interactive Tree Of Life (iTOL) v4: recent updates and new developments. *Nucleic Acids Res* **47**, W256-W259 (2019).

4. Arndt D*, et al.* PHASTER: a better, faster version of the PHAST phage search tool. *Nucleic Acids Res* **44**, W16-21 (2016).

5. Tong T*, et al.* Occurrence, evolution, and functions of DNA phosphorothioate epigenetics in bacteria. *Proc Natl Acad Sci U S A* **115**, E2988-E2996 (2018).

6. Yang Y*, et al.* DNA Backbone Sulfur-Modification Expands Microbial Growth Range under Multiple Stresses by its anti-oxidation function. *Sci Rep* **7**, (2017).

7. Wu X*, et al.* Epigenetic competition reveals density-dependent regulation and target site plasticity of phosphorothioate epigenetics in bacteria. *PNAS* **117**, 14322–14330 (2020).

8. Robinson MD, McCarthy DJ, Smyth GK. edgeR: a Bioconductor package for differential expression analysis of digital gene expression data. *Bioinformatics* **26**, 139-140 (2010).

9. Baba T*, et al.* Construction of *Escherichia coli* K-12 in-frame, single-gene knockout mutants: the Keio collection. *Mol Syst Biol* **2**, 2006.0008 (2006).

10. Blattner FR*, et al.* The complete genome sequence of *Escherichia coli* K-12. *Science* **277**, 1453-1462 (1997).

11. Jian H, Xiao X, Wang F. Role of filamentous phage SW1 in regulating the lateral flagella of *Shewanella piezotolerans* strain WP3 at low temperatures. *Appl Environ Microbiol* **79**, 7101-7109 (2013).

12. Yang X, Jian H, Wang F. pSW2, a novel low-temperature-inducible gene expression vector based on a filamentous phage of the deep-sea bacterium *Shewanella piezotolerans* WP3. *Appl Environ Microbiol* **81**, 5519-5526 (2015).

13. Jian H, Xu J, Xiao X, Wang F. Dynamic modulation of DNA replication and gene transcription in deep-sea filamentous phage SW1 in response to changes of host growth and temperature. *PLoS One* **7**, e41578 (2012).

14. Wang L, *et al.* Phosphorothioation of DNA in bacteria by *dnd* genes. *Nat Chem Biol* **3**, 709-710 (2007).

15. Xie X, *et al.* Phosphorothioate DNA as an antioxidant in bacteria. *Nucleic Acids Res* **40**, 9115-9124 (2012).

16. An X, *et al.* A Novel Target of IscS in *Escherichia coli*: Participating in DNA phosphorothioation. *PLoS One* **7**, e51265 (2012).
