# Supplementary material for: The origin and impeded dissemination of the DNA phosphorothioation system in prokaryotes
Source: Nat Commun. 2021 Nov 4;12:6382. doi: 10.1038/s41467-021-26636-7 (PMC8569181; doi:10.1038/s41467-021-26636-7)
Supplement: Supplementary file 2 — Description of Additional Supplementary Files [file 41467_2021_26636_MOESM2_ESM.docx]

**Description of Additional Supplementary Files**

**File Name:** Supplementary Data 1

**Description:** Detailed information of the occurrence and distribution of dnd genes and gene clusters in prokaryotic genomes.

**File Name:** Supplementary Data 2

**Description:** Detailed information of the prophages and the repressors & regulators they contain.

**File Name:** Supplementary Data 3

**Description:** Statistics of the correlation analysis between the abundance of dnd genes/gene clusters and prophages.

**File Name:** Supplementary Data 4

**Description:** Detailed information of the PT modifications in WP3NR genome.
