# Supplementary material for: The origin and impeded dissemination of the DNA phosphorothioation system in prokaryotes
Source: Nat Commun. 2021 Nov 4;12:6382. doi: 10.1038/s41467-021-26636-7 (PMC8569181; doi:10.1038/s41467-021-26636-7)
Supplement: Supplementary file 7 — Reporting Summary [file 41467_2021_26636_MOESM7_ESM.pdf]

nature portfolio

Corresponding author(s): Xiang Xiao
Last updated by author(s): Oct 15, 2021

Reporting Summary

Nature Portfolio wishes to improve the reproducibility of the work that we publish. This form provides structure for consistency and transparency in reporting. For further information on Nature Portfolio policies, see our Editorial Policies and the Editorial Policy Checklist.

Statistics

For all statistical analyses, confirm that the following items are present in the figure legend, table legend, main text, or Methods section.

n/a	Confirmed	
☐	☒	The exact sample size (*n*) for each experimental group/condition, given as a discrete number and unit of measurement
☐	☒	A statement on whether measurements were taken from distinct samples or whether the same sample was measured repeatedly
☐	☒	The statistical test(s) used AND whether they are one- or two-sided<br>*Only common tests should be described solely by name; describe more complex techniques in the Methods section.*
☒	☐	A description of all covariates tested
☒	☐	A description of any assumptions or corrections, such as tests of normality and adjustment for multiple comparisons
☐	☒	A full description of the statistical parameters including central tendency (e.g. means) or other basic estimates (e.g. regression coefficient) AND variation (e.g. standard deviation) or associated estimates of uncertainty (e.g. confidence intervals)
☐	☒	For null hypothesis testing, the test statistic (e.g. *F*, *t*, *r*) with confidence intervals, effect sizes, degrees of freedom and *P* value noted<br>*Give P values as exact values whenever suitable.*
☒	☐	For Bayesian analysis, information on the choice of priors and Markov chain Monte Carlo settings
☒	☐	For hierarchical and complex designs, identification of the appropriate level for tests and full reporting of outcomes
☐	☒	Estimates of effect sizes (e.g. Cohen's *d*, Pearson's *r*), indicating how they were calculated

Our web collection on statistics for biologists contains articles on many of the points above.

Software and code

Policy information about availability of computer code

Data collection	No software was used to collect data.
Data analysis	DIAMOND v0.9.25; VIBRANT v1.2.1; MAFFT v7.313; trimAl v1.2; RAxML v8.0; GTDB-Tk v1.3.0; FastTree v2.1.10; MEGA X v10.2.2; iTOL v4; SciPy v1.0; R v3.5.3; Primer Express v3.0.1; 7500 System SDS v2.0.6; Biacore Insight Evaluation v1.0; SMRT Analysis Suite v1.3; fastq v0.19.7; HISAT v2.1.0; RSEM v1.3.1; DEGseq v1.36.0; edgeR v3.20.2

For manuscripts utilizing custom algorithms or software that are central to the research but not yet described in published literature, software must be made available to editors and reviewers. We strongly encourage code deposition in a community repository (e.g. GitHub). See the Nature Portfolio guidelines for submitting code & software for further information.

Data

Policy information about availability of data

All manuscripts must include a data availability statement. This statement should provide the following information, where applicable:

- Accession codes, unique identifiers, or web links for publicly available datasets
- A description of any restrictions on data availability
- For clinical datasets or third party data, please ensure that the statement adheres to our policy

All bacterial and archaeal assembled genomes (n=22,668) used in this study were collected from the NCBI RefSeq database (Release 202, https://www.ncbi.nlm.nih.gov/refseq/). The Dnd protein sequenc-es were retrieved from the KEGG Orthology (KO) database (https://www.genome.jp/kegg/ko.html). All the detailed information of the identified dnd genes, gene clusters and prophages are described and available publicly in Supplementary Data 1 and 2. The epigenomic sequencing data of S. piezotolerans WP3NR/Dnd generated using the PacBio RSII platform are available in Figshare [https://figshare.com/articles/-dataset/SMRT_sequencing_data_of_Shewanella_piezotolerans_WP3NR_Dnd/16809943]. The transcriptomic data from the current study have been deposited in the NCBI SRA under project ID PRJNA565632 [https://www.ncbi.nlm.nih.gov/sra/?term=PRJNA565632]. Source data are provided with this paper.

Field-specific reporting

Please select the one below that is the best fit for your research. If you are not sure, read the appropriate sections before making your selection.

☒ Life sciences ☐ Behavioural & social sciences ☐ Ecological, evolutionary & environmental sciences

For a reference copy of the document with all sections, see nature.com/documents/nr-reporting-summary-flat.pdf

Life sciences study design

All studies must disclose on these points even when the disclosure is negative.

Sample size	To avoid the effect of low genomic quality on the analysis of the distribution of the dnd system and prophages, only the assembly level'of complete genome or chromosome was selected. At the time when we retrieved the RefSeq genome database, a total of 22,280 bacterial and 388 archaeal genomes were available.
Data exclusions	For clarity, only the bacterial orders and archaeal phyla with ≥30 high-quality genomes were included in the phylogenetic tree (Fig. 1c). To facilitate statistical analysis, only the genus and species with ≥30 genomes carrying at least 1 prophage or dnd gene cluster were included in the correlation analyses (Fig. 3).
Replication	Normally, values represent the mean ± SD of triplicate assays in this study, and all attempts at replication were successful.
Randomization	Randomization was not applicable to this study. All complete prokaryotic genomes in NCBI RefSeq were explicitly searched for dnd systems and prophages, and randomization was not relevant for these non-biased analyses.
Blinding	Blinding was not applicable to this study. Knowledge of the dnd system and prophage was essential for analysis. The blinding was not relevant, and the exclusion of it had no effect on the accuracy and reliability of the results.

Reporting for specific materials, systems and methods

We require information from authors about some types of materials, experimental systems and methods used in many studies. Here, indicate whether each material, system or method listed is relevant to your study. If you are not sure if a list item applies to your research, read the appropriate section before selecting a response.

Materials & experimental systems

n/a	Involved in the study	
☒	☐	Antibodies
☒	☐	Eukaryotic cell lines
☒	☐	Palaeontology and archaeology
☒	☐	Animals and other organisms
☒	☐	Human research participants
☒	☐	Clinical data
☒	☐	Dual use research of concern

Methods

n/a	Involved in the study	
☒	☐	ChIP-seq
☒	☐	Flow cytometry
☒	☐	MRI-based neuroimaging
